# MXRA7 attenuates sperm injury from exercise-induced fatigue via suppression of epididymal inflammation

**DOI:** 10.1016/j.redox.2026.104301

**Published:** 2026-07-14

**Authors:** Kunyang Tang, Xiaocui Jiang, Yanyan Zhou, Xin Hu, Jiasen Liu, Donghui Huang, Xiaoming Yu, Min Zhao, Ying Liu, Jigang Cao, Zhipeng Fang, Min Xiao

**Affiliations:** a Department of Physical Education and Health, Hubei University of Chinese Medicine, Wuhan, 430065, China; b Laboratory Animal Center, Hubei University of Chinese Medicine, Wuhan, 430065, China; c Basic Medical College, Hubei University of Chinese Medicine, Wuhan, 430065, China; d Hubei Shizhen Laboratory, Wuhan, 430065, China; e Institute of Reproduction Health Research, Tongji Medical College, Huazhong University of Science and Technology, Wuhan, 430030, China

**Keywords:** Matrix-remodeling associated 7, Exercise-induced fatigue, Oxidative stress, Pyroptosis, Epididymis

## Abstract

Exercise-induced fatigue (EIF) is closely associated with male reproductive dysfunction, but the underlying mechanism remains unclear. This study aimed to explore whether EIF induces oxidative stress and epididymal inflammation, and to clarify the protective role and regulatory pathway of matrix remodeling-associated 7 (MXRA7). Clinical samples from EIF volunteers and an EIF mouse model were used to detect oxidative stress, inflammation, and sperm quality. Mouse caput epididymal PC-1 cells and cauda epididymal DC-2 cells were cultured to establish inflammatory injury models. MXRA7 expression, localization, and function were analyzed by transcriptomic analysis, gene knockdown/overexpression, Western blotting, immunofluorescence, co-immunoprecipitation, and in vitro phosphorylation assays. The results showed that EIF significantly elevated systemic and epididymal oxidative stress and inflammatory responses in both humans and mice, accompanied by impaired sperm motility and epididymal dysfunction. MXRA7 was highly expressed in epididymal epithelial cells, especially in the cauda epididymis, and its expression was negatively correlated with the severity of inflammation. MXRA7 knockdown aggravated inflammatory injury in DC-2 cells, whereas MXRA7 overexpression suppressed oxidative stress, inflammatory factor release, and NF-κB signaling activation. Mechanistically, protein kinase C alpha (PKCα) mediated the expression and phosphorylation of MXRA7, and MXRA7 further inhibited the NF-κB pathway to alleviate epididymal inflammation. In addition, MXRA7 expressed by cauda epididymal epithelial cells directly protected sperm from inflammatory damage. In conclusion, EIF impairs sperm function by triggering epididymal oxidative stress and inflammatory injury. MXRA7, regulated by PKCα-mediated phosphorylation, serves as a key protective factor that attenuates EIF-induced epididymal inflammation via inhibiting the NF-κB signaling pathway. This study provides a novel target for the prevention and treatment of reproductive damage caused by excessive exercise.

## Highlights

• Exercise-induced fatigue triggers epididymal inflammation, causing sperm damage.
• MXRA7 shows segment-specific expression in the epididymis.
• MXRA7 inhibits NF-κB pathways to attenuate epididymal inflammation.
• PKCα mediates the expression and phosphorylation of MXRA7.
• MXRA7 expressed by cauda epididymis attenuates inflammation-induced sperm damage.

## Graphical abstract

**Figure:**
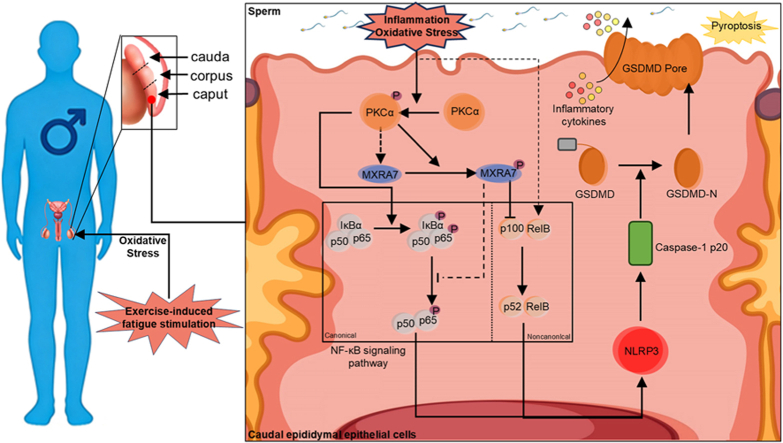

## 1 Introduction

Exercise-induced fatigue (EIF) is a condition characterized by a transient decline in physiological function caused by excessive or inappropriate physical activity [1]. Transient and mild EIF is a normal physiological adaptive response that resolves spontaneously without specific intervention; however, severe and prolonged EIF can cause systemic dysfunction and even induce organic diseases [2], with damage to the male reproductive system being of particular concern. Accumulating evidence, including our previous studies, has demonstrated that EIF is closely associated with the development of oligoasthenospermia in men [[2], [3], [4], [5]], yet the underlying molecular mechanisms remain incompletely understood. EIF can trigger systemic oxidative stress and inflammatory responses, and the male reproductive system is particularly vulnerable to damage caused by oxidative stress and inflammation [[6], [7], [8]]. Meanwhile, mechanical friction on reproductive organs during exercise, as well as systemic pressure and stress responses, can further exacerbate reproductive system damage [5,9]. Therefore, we hypothesize that EIF-induced sperm quality impairment may be closely related to local oxidative stress and inflammatory responses in the reproductive system.

The epididymis is a highly convoluted tubular organ connecting the testis and vas deferens, which is divided into three segments (caput, corpus, and cauda) with distinct structural and functional heterogeneity, and plays an irreplaceable role in sperm maturation, storage, and protection. Spermatozoa freshly released from the testis are immotile and lack fertilizing potential; they must undergo a maturation process in the microenvironment maintained by epididymal epithelial cells to acquire progressive motility and fertilizing capacity [10]. Our study suggests that EIF may induce oxidative stress and epididymal inflammation, thereby impairing sperm quality [11]. Epidemiological data show that the global incidence of epididymitis is approximately 400 cases per 100,000 men annually [12], and it is one of the leading causes of persistent oligoasthenospermia and azoospermia [13]. It is traditionally believed to be caused by ascending bacterial infection of the genitourinary tract. However, recent studies have identified sterile inflammation as an important etiological factor, particularly in cases associated with physical stress and trauma [14,15]. Emerging evidence suggests that the inflammatory activity in the caput epididymidis is significantly higher than that in the cauda during epididymitis, yet the molecular basis for this segment-specific difference in inflammatory susceptibility remains unclear [10]. Furthermore, our preliminary studies have demonstrated that EIF can induce pyroptosis in epididymal cells—a highly pro-inflammatory form of programmed cell death [16]. Pyroptosis is initiated by inflammasome assembly, which leads to mitochondrial dysfunction [17] and subsequent activation of gasdermin D (GSDMD). Activated GSDMD forms ring-shaped pores in the plasma membrane, resulting in cell lysis and the release of large amounts of pro-inflammatory cytokines [18]. Pyroptosis of epididymal epithelial cells, which are in direct contact with spermatozoa, is likely to disrupt the homeostasis of the epididymal microenvironment and cause direct damage to sperm.

Matrix remodeling-associated 7 (MXRA7) is a secreted functional protein that acts as an autocrine/paracrine factor involved in multiple biological processes [19]. Although research on MXRA7 is still limited, scattered studies have revealed its significant roles in inflammation and immune regulation [20], including regulatory interactions with core inflammatory pathways such as NF-κB [21], as well as involvement in cell proliferation, differentiation, and wound healing [19]. Our preliminary studies have demonstrated that MXRA7 is differentially expressed in the caput, corpus, and cauda epididymidis, and its expression level is significantly negatively correlated with the degree of inflammation, suggesting that MXRA7 may be a key molecule mediating segment-specific inflammatory responses in the epididymis. Based on the above background, we propose the hypothesis that EIF induces epididymal oxidative stress and inflammation, leading to pyroptosis of epididymal epithelial cells and subsequent sperm damage, while MXRA7 alleviates EIF-induced sperm injury by inhibiting epididymal inflammation. In this study, we combined clinical samples, in vivo animal models, and in vitro cell experiments to systematically investigate the role and molecular mechanism of MXRA7 in EIF-related inflammatory injury, aiming to provide novel therapeutic targets and theoretical basis for the prevention and treatment of exercise-induced reproductive damage.

## 2 Materials and methods

### 2.1 Reagents

Vitamin C, lipopolysaccharide (LPS), phorbol 12-myristate 13-acetate (PMA), BIM 1 (Orileaf, Shanghai, China); Tunel, IL-6, IL-1β, and TNF-α assay kits; 8-OhdG; FITC-labeled *Arachis hypogaea* agglutinin; Anti-MXRA7 antibody, Anti-p-PKCα (Ser657/Tyr658) Antibody (Sigma-Aldrich (Shanghai), Shanghai, China); Recombinant mouse MXRA7 (rMXRA7) protein (Cusabio, Wuhan, China); Caspase 1 p20 (Cleaved Asp296) Polyclonal Antibody (Thermo Fisher Scientific, Waltham, USA); Anti-Hexokinasel antibody; Anti-COX IV antibody; Anti-α-Tubulin antibody; Anti-NLRP3 antibody, Anti-Caspase-1 antibody, Anti-GSDMD antibody, Anti-p–NF–κB p65 antibody, Anti–NF–κB p65 antibody, Anti-p–NF–κB IκBα antibody, Anti–NF–κB IκBα antibody, Anti–NF–κB p52/p100 antibody, Anti-PKCα antibody, Anti-pan-PKC antibody, HRP-conjugated Goat Anti-Rabbit IgG(H + L), (Sanying, Wuhan, China); Phos-tag SDS-PAGE (FUJIFILM Wako, Osaka, Japan); DAPI staining reagent (Servicebio, Wuhan, China).

### 2.2 Human subjects

Human semen and serum samples were collected from volunteers aged 25–40 years at the Guanggu Campus of Hubei Provincial Hospital of Traditional Chinese Medicine. All volunteers attended the hospital either voluntarily or upon invitation for reproductive medicine diagnosis or treatment, and were all native speakers of simplified Chinese. Sample evaluation was performed in accordance with the principles outlined in the 6th edition of the World Health Organization (WHO) Laboratory Manual for the Examination and Processing of Human Semen (World Health Organization, 2021). All experiments were conducted in compliance with the Declaration of Helsinki of the World Medical Association. The study was approved by the Institutional Ethics Committee (approval No.: HBZY2023-C10-02), and written informed consent was obtained from each participant.

We initially collected sperm data from 169 male participants, and 45 volunteers remained after the implementation of inclusion and exclusion criteria, including 15 patients with exercise-induced fatigue (EIF) who consented to both sperm and serum sample collection (among them, 4 had high-intensity EIF [EIF-H]), and 30 healthy volunteers, among whom 15 agreed to serum sample donation. The inclusion criteria for EIF patients were defined as follows: 1) Male subjects aged 18–45 years who visited the hospital voluntarily or upon invitation for sperm quality testing due to reproductive health consultation and fertility preparation needs, with signed informed consent and willingness to cooperate with the study procedures. 2)A confirmed history of EIF exposure within 1 week prior to testing: for regular exercisers, the intensity of a single exercise session was increased by more than 50% relative to their routine level for 3 consecutive days; for irregular exercisers, sudden high-intensity exercise was performed for more than 3 consecutive days. 3)A score of ≥7 on the Fatigue Severity Scale (FS-14) and a score of ≥15 on the Borg Rating of Perceived Exertion (RPE) scale immediately after exercise; persistent fatigue symptoms were reported daily within the last 2 weeks, which were exacerbated after exercise, could not be completely relieved even after more than 30 min of rest, and persisted until the time of testing. 4)Provision of a complete semen sample with a volume of ≥1.5 mL, without missing baseline data. Among them, those with an FS-14 score ≥10, an RPE score ≥17, and no significant relief after resting for more than 1 h were defined as EIF-H.

The inclusion criteria for healthy volunteers were as follows: 1) Male subjects aged 18–45 years who underwent sperm quality testing at the hospital during the same period, with an age difference of ±5 years relative to the EIF patient group, and who provided informed consent and voluntarily agreed to participate in the study. 2) No history of EIF exposure within the preceding two weeks; a score of ≤3 on the Fatigue Severity Scale (FS-14) and a score of ≤11 on the Borg Rating of Perceived Exertion (RPE) scale immediately after exercise. 3) Normal sperm parameters and no history of definite diseases, including no medical history of reproductive system disorders, endocrine system disorders, or chronic systemic diseases. 4) Compliance with the same semen sample collection standards as the EIF patient group, with complete and traceable baseline data.

Exclusion criteria were as follows: 1) A history of organic diseases or surgical interventions related to the reproductive system. 2) Presence of systemic diseases, including endocrine/metabolic disorders (e.g., diabetes mellitus, thyroid dysfunction), autoimmune diseases, chronic infectious diseases (e.g., tuberculosis, human immunodeficiency virus (HIV) infection), and hepatic or renal insufficiency. 3) Exposure to interfering medications or lifestyle factors: use of drugs affecting sperm quality (e.g., antidepressants, hormonal agents) within the preceding 3 months; smoking ≥10 cigarettes per day for ≥1 consecutive year; alcohol dependence or substance abuse; or administration of hormone supplements and high-dose antioxidants. 4) Presence of confounding factors for fatigue diagnosis: fatigue caused by non-exercise related etiologies (e.g., anemia, sleep duration <6 h per day for ≥1 consecutive month); confirmed diagnosis of depression/anxiety with ongoing medication; or incomplete or logically inconsistent completion of study scales. 5) Other confounding factors: chromosomal abnormalities, non-standardized semen sample collection, or missing baseline data.

### 2.3 Collection of human samples

Semen samples were obtained via masturbation following 3–5 days of sexual abstinence. After liquefaction at 37 °C for 30 min, aliquots of the semen were taken for subsequent analysis. The remaining liquefied semen was centrifuged at 400 × g for 5 min (CR21G centrifuge, Hitachi, Tokyo, Japan) at room temperature to separate spermatozoa from seminal plasma [22]. The supernatant seminal plasma was transferred and subjected to three consecutive rounds of centrifugation at 700 × g for 5 min at 4 °C; the resulting precipitates were discarded, and the purified seminal plasma was stored at −80 °C. The sperm pellets were resuspended in phosphate-buffered saline (PBS) and washed three times, followed by fixation in 4% PFA at room temperature for 5 min. The fixed spermatozoa were then washed again by centrifugation at 700 × g for 5 min in PBS, spread onto air-dried glass slides, and stored at −20 °C.

Serum samples were collected from fasting subjects (8–12 h of food and water deprivation) on the same day as semen sample collection. After disinfecting the venipuncture site, 3–5 mL of venous blood was drawn and allowed to clot at 25 °C for 30 min. The clotted blood was centrifuged at 3000 × g for 10 min at 4 °C to separate serum. The upper serum layer was aspirated and immediately stored at −80 °C. All procedures were completed within 2 h after blood collection.

### 2.4 Transcriptomic analysis (RNA-seq)

Transcriptome data, including 3 samples from GSE145443 and 6 samples from GSE199903, were downloaded from the NCBI Gene Expression Omnibus (GEO) public database (https://www.ncbi.nlm.nih.gov/geo/info/datasets.html) for transcriptomic analysis. First, the expression profiles were imported and processed using the Seurat R package. Cells were filtered based on three metrics per cell: the total number of unique molecular identifiers (UMIs), the number of expressed genes, the percentage of mitochondrial gene reads, and the percentage of ribosomal gene reads. Outliers were defined as values deviating by more than three median absolute deviations (MADs) from the median value. In general, cells with excessively high total UMI counts or numbers of expressed genes are considered doublets, whereas cells with excessively high percentages of mitochondrial or ribosomal gene reads are regarded as low-quality cells that are either undergoing apoptosis or have degraded into cellular debris.

The NormalizeData function was used to standardize the raw data, followed by calculation of cell cycle scores via the CellCycleScoring function, identification of highly variable genes using FindVariableFeatures, and normalization of the data with ScaleData—this step also eliminated confounding effects of mitochondrial genes, ribosomal genes, and cell cycle phase on subsequent analyses. Linear dimensionality reduction of the expression matrix was performed using RunPCA, and the top principal components (PCs) were selected for downstream analyses. Batch effects were removed with Harmony, an algorithm that iteratively clusters similar cells from different batches in the PCA space while preserving batch diversity within each cluster. Non-linear dimensionality reduction was conducted via RunUMAP (Uniform Manifold Approximation and Projection, UMAP). Cell neighbors were identified using FindNeighbors, and cells were partitioned into distinct clusters with FindClusters. Cell annotation was performed by identifying cell types and their corresponding marker genes present in the target tissue; this was primarily achieved by querying the CellMarker database and reviewing relevant literature, with automated annotation using the SingleR software as a supplementary approach.

For RNA-seq data analysis, lowly expressed genes were filtered out with the criterion of retaining only those genes with a count >1 in ≥10% of the samples. The raw count data were then normalized using the trimmed mean of M-values (TMM) method via the edgeR package and subsequently transformed into log2-counts per million (log2-CPM). Based on the median expression level of MXRA7 (Entrez ID: 439921), the samples were stratified into a MXRA7 high-expression group and a MXRA7 low-expression group. Differential expression analysis was performed using the limma package to calculate the log2 fold change (logFC) of each gene. The resulting genes were ranked by logFC values to generate a ranked gene list for gene set enrichment analysis (GSEA). The annotated gene sets of version 7.0 downloaded from the Molecular Signatures Database (MsigDB) was used as the background gene set for pathway enrichment analysis among different groups, and the significantly enriched gene sets (adjusted p-value <0.05) were sorted according to their normalized enrichment scores (NES). GSEA was further conducted using two gene sets defined by Entrez IDs: one derived from the NF-κB pathway in the Kyoto Encyclopedia of Genes and Genomes (KEGG) database, and the other a custom gene set corresponding to the canonical and non-canonical NF-κB pathways. Significance was assessed by 1000 permutation tests, and GSEA plots illustrating the regulatory effect of MXRA7 on the NF-κB pathway were generated using the enrichplot package.

### 2.5 Animal models and interventions

Specific-pathogen-free (SPF) male ICR mice (weighing 30–32 g, purchased from the Hubei Provincial Center for Experimental Animals) were subjected to a 7-day acclimatization period prior to the experiment. The mice were randomly divided into three groups: the control group (CON), the exercise-induced fatigue group (EIF), and the vitamin C intervention group (Vitamin C group), with 15 mice per group. The EIF animal model was established as described previously [23], using a 4-week consecutive weight-loaded swimming training protocol. The swimming tank used for training had a water depth of 50 cm and a water temperature of (31 ± 2) °C. During each training session, the mice were placed on the water surface, and a glass rod was gently stirred beside them to guide the mice to swim. The training lasted for 4 weeks, with the following progressive loading regimen: no weight was applied in the first week, 2% of the body weight was used as the load in the second week, 4% in the third week, and 5% in the fourth week. Each training session was terminated when the mice reached exhaustion, which was defined as the mouse failing to surface within 3 s after sinking. After the 4-week weight-loaded swimming training, all mice underwent a single session of unloaded exhaustive swimming, and the exhaustive swimming time was recorded for each mouse. Except for the CON group, mice in the other two groups received daily weight-loaded swimming training. During the 3rd–4th weeks of weight-loaded swimming training, mice in the Vitamin C group were administered vitamin C via gavage at a daily dose of 40 mg/kg body weight. The gavage dose of vitamin C was based on the intervention dose for weight-loaded swimming mice described in Ref. [24]. The body weight of each mouse was recorded daily throughout the modeling period. Following the final unloaded exhaustive swimming session, the mice were retrieved, dried thoroughly, anesthetized, and subjected to blood collection. The collected blood samples were allowed to stand for 1 h, then centrifuged at 3000 r/min for 10 min at 4 °C. The serum was separated and stored at −80 °C. The left gastrocnemius muscle and epididymides from both sides were harvested and preserved. The weight of the epididymides was measured immediately. A portion of the epididymides was punctured, placed in pre-warmed PBS at 37 °C, and incubated in a 37 °C water bath for subsequent detection. Seminal plasma and spermatozoa were separated, and sperm smears were prepared as previously described [25]. All experimental procedures were strictly performed in accordance with the Guidelines for the Care and Use of Laboratory Animals and were approved by the Institutional Animal Care and Use Committee (IACUC) of Hubei University of Chinese Medicine (Approval No.: HUCMS55714520).

### 2.6 Determination of testosterone, BUN, LA, LDH, IL-1β, IL-6 and TNF-α

Liquid samples derived from humans or mice were assayed using enzyme-linked immunosorbent assay (ELISA) kits specific to the corresponding species, targeting testosterone, blood urea nitrogen (BUN), lactic acid (LA), lactate dehydrogenase (LDH), interleukin-1β (IL-1β), interleukin-6 (IL-6), and tumor necrosis factor-α (TNF-α) (MEIMIAN, Jiangsu, China). All procedures were performed strictly in accordance with the manufacturers’ instructions.

### 2.7 Malondialdehyde (MDA) measurement

The level of MDA, a marker of lipid peroxidation, was detected using the thiobarbituric acid reactive substances (TBARS) assay (Beyotime Biotechnology, Shanghai, China). Briefly, 100 μL of sample supernatant was mixed with 100 μL of 0.2% thiobarbituric acid (TBA) solution (dissolved in glacial acetic acid) and 5 μL of 0.01% BHT. The mixture was sealed and incubated in a 95 °C water bath for 30 min, then immediately placed in an ice bath to terminate the reaction. After centrifugation at 12,000 × g for 10 min at 4 °C, the fluorescence intensity of the supernatant was measured at an excitation wavelength of 532 nm and an emission wavelength of 553 nm using a microplate reader. The MDA concentration of each sample was calculated according to the standard curve prepared with 1,1,3,3-tetraethoxypropane (TEP), and the results were normalized to the protein concentration and expressed as nmol/mL.

### 2.8 Superoxide dismutase (SOD) activity assay

The total SOD activity was determined using the xanthine oxidase method (WST-8) (Beyotime Biotechnology, Shanghai, China), which reflects the intrinsic antioxidant capacity of the sample. Briefly, 20 μL of sample supernatant was added to the reaction system containing xanthine substrate, xanthine oxidase, and chromogenic reagent, mixed well, and incubated in a 37 °C water bath for 20 min. The absorbance of each well was measured at 550 nm using a microplate reader. One unit (U) of SOD activity was defined as the amount of enzyme required to achieve 50% inhibition of the superoxide anion-mediated chromogenic reaction in 1 mL of the reaction system. The results were normalized to the protein concentration and expressed as U/mL.

### 2.9 Determination of reduced glutathione (GSH) and oxidized glutathione (GSSG)

The levels of total GSH and GSSG were measured using the DTNB-glutathione reductase recycling assay (Beyotime Biotechnology, Shanghai, China). Briefly, the sample supernatant was mixed with 5% sulfosalicylic acid at a ratio of 5:1 to precipitate proteins, and centrifuged at 10,000 × g for 10 min at 4 °C to collect the supernatant. For total GSH detection, the supernatant was added to the reaction system containing DTNB, glutathione reductase, and NADPH, and the absorbance at 412 nm was continuously monitored at 37 °C. For GSSG detection, the supernatant was pretreated with 2-vinylpyridine for 60 min at room temperature to scavenge free GSH, followed by the same detection procedure as total GSH. The concentration of free GSH was calculated as: free GSH = total GSH - 2 × GSSG. The GSH/GSSG ratio was then calculated, and the results were normalized to the protein concentration and expressed as μmol/mL.

### 2.10 JC-1 mitochondrial membrane potential detection

Flow cytometry was conducted with the JC-1 Mitochondrial Membrane Potential Detection Kit (Beyotime Biotechnology, Shanghai, China). Briefly, in accordance with the manufacturer's instructions, the sperm suspension was filtered through a 200-mesh sieve, and approximately 10^5^ to 10^6^ cells were collected and centrifuged at 300 × g for 5 min. After discarding the supernatant, the cell pellet was resuspended in 500 μL of JC-1 working solution and incubated at 37 °C for 20 min in the dark. Following centrifugation and washing, the cells were resuspended with 1× JC-1 Assay Buffer and subsequently detected by flow cytometry [26].

### 2.11 Analysis of sperm parameters using an automatic sperm analyzer

After complete liquefaction of human or murine semen samples, 5 μL of semen was pipetted onto a pre-warmed specialized glass slide. Sperm parameters were analyzed using the BEION Sperm Quality Analysis and Management System (BEION, Shanghai, China) at 37 °C. The system captured six randomly selected microscopic fields (25 frames per second; 25 images per field), with all parameters analyzed automatically by the software. The analyzed parameters included sperm concentration (×10^6^ cells/mL), sperm motility (%), progressive motility (%), average path velocity (VAP), curvilinear velocity (VCL), straight-line velocity (VSL), amplitude of lateral head displacement (ALH), and beat-cross frequency (BCF).

### 2.12 Immunofluorescence

For cell and sperm staining: Frozen fixed sperm smears were thawed (direct exposure to ambient air was avoided during thawing to prevent contamination by condensation) or pre-prepared cell climbing slides were retrieved. The samples were permeabilized with 1% Triton X-100 and then blocked with 5% bovine serum albumin (BSA) for 1 h. Pre-adsorbed primary antibodies were added to the slides, followed by incubation at 4 °C for 14–16 h. After washing the slides five times with phosphate-buffered saline with Tween-20 (PBST), secondary antibodies were applied and incubated for 1 h. Nuclei were stained with 4′,6-diamidino-2-phenylindole (DAPI) for 5 min, and images were visualized under a A1R SI fluorescence microscope (Nikon, Tokyo, Japan) [25]. For animal tissue staining: Epididymal tissues were harvested and fixed in a universal fixative for 48 h at room temperature, followed by paraffin embedding and sectioning. Paraffin sections were baked, dewaxed in a graded series of xylene and ethanol solutions, and then subjected to hydration and antigen retrieval. Sections were incubated with a solution containing 15% BSA and 0.5% Triton X-100 at 37 °C for 1 h. Primary antibodies (or a mixture of the two primary antibodies) were then added at a dilution of 1:50 and incubated at 4 °C overnight. After rinsing with phosphate-buffered saline (PBS), secondary antibodies (or a mixture of the corresponding fluorescent secondary antibodies) were added at a dilution of 1:1000 and incubated at 37 °C for 30 min. Following PBS rinses, cells were stained with specific dyes. Immediately after the final PBS rinse, sections were mounted using an anti-fluorescence quenching mounting medium, and fluorescence images were captured under a fluorescence microscope.

### 2.13 Observation of sperm acrosome

The processing method for semen samples used in acrosome detection was performed as described in Study [27] with minor modifications. Briefly, semen samples were incubated at 37 °C for 2 h to induce capacitation, followed by a further 1 h incubation at 37 °C with the addition of progesterone (5 μM). The samples were then centrifuged at 700 × g for 5 min at room temperature, and resuspended in a hypotonic swelling medium (PBS: ddH_2_O = 1:10) for 1 h at 37 °C. After an additional washing step, the spermatozoa were resuspended and fixed in 4% PFA for 5 min at room temperature, then washed again by centrifugation at 700 × g for 5 min in PBS, and finally spread onto air-dried glass slides. For detection, the slides were incubated with 1 mg/mL FITC-labeled *Arachis hypogaea* agglutinin (PNA) in PBS at 4 °C for 20 min in the dark. The slides were rinsed with PBS, incubated with 5 mg/mL DAPI in ddH_2_O for 10 s at room temperature, washed again with PBS, air-dried, and imaged under a fluorescence microscope.

### 2.14 Hypo-osmotic swelling (HOS) test

The protocol was performed as described in Studies [28,29] with minor modifications. Processed sperm samples were gently mixed with 150 μL of hypo-osmotic swelling (HOS) solution (containing 1.5 mM fructose and 1.5 mM sodium citrate), followed by incubation at 37 °C for 30 min. Subsequently, 200 spermatozoa were observed under a E100 microscope (Nikon, Tokyo, Japan), and the number of abnormal spermatozoa (i.e., the proportion of sperm exhibiting tail swelling) was counted.

### 2.15 Histological morphology observation

Fixed gastrocnemius and epididymal tissues were subjected to dehydration, clearing, and wax infiltration prior to paraffin embedding. Sections with a thickness of 5 μm were cut, stained with hematoxylin and eosin (H&E), mounted with neutral resin, and observed under a light microscope.

### 2.16 Immunohistochemistry

Fixed epididymal tissues were subjected to dehydration, clearing, and wax infiltration, followed by paraffin embedding. Paraffin sections were first dewaxed and rehydrated. The staining procedure included the following steps: antigen retrieval, treatment with 3% hydrogen peroxide, demarcation of the target area, serum blocking, incubation with primary antibodies at 4 °C overnight, incubation with secondary antibodies at room temperature, color development, and counterstaining with hematoxylin. Finally, the sections were dehydrated and mounted before microscopic examination.

### 2.17 Transmission electron microscopy (TEM) observation

After mice were anesthetized and sacrificed, bilateral epididymal tissues were quickly isolated and placed in pre-cooled 2.5% glutaraldehyde fixative. The tissues were cut into small pieces of approximately 1 mm^3^ and fixed at 4°C for 24 h. After fixation, the tissue blocks were rinsed with PBS three times; then transferred to 1% osmium tetroxide fixative for post-fixation at 4°C for 2 h, followed by another three rinses with PBS. The samples were then dehydrated using a graded ethanol series and finally replaced with anhydrous acetone twice. Subsequently, the samples were infiltrated and embedded in epoxy resin, sectioned, and stained with uranyl acetate and lead citrate. The ultrastructure of epididymal epithelial cells was observed using an HT7800 transmission electron microscope (Hitachi, Tokyo, Japan).

### 2.18 Cell culture

The conditionally immortalized murine distal epididymal epithelial cell line (DC-2) and proximal epididymal epithelial cell line (PC-1) were purchased from Shanghai Yaji Biotechnology Co., Ltd (Shanghai, China). These cell lines were isolated from transgenic mice harboring a temperature-sensitive simian virus 40 large T tumor antigen. The culture conditions were as follows: the cells were cultured in complete Iscove's Modified Dulbecco's Medium (IMDM) supplemented with 10% fetal bovine serum (FBS) and 1% penicillin/streptomycin [9], in a humidified incubator at 37 °C with 5% CO_2_. For pyroptosis induction [30], the cells were seeded into 6-well plates at a density of 1 × 10^6^ cells/mL. After 24 h of culture, the cells were incubated with lipopolysaccharide (LPS) at a concentration of 1 μg/mL for 5 h, followed by treatment with adenosine triphosphate (ATP) at 5 mM for 12 h to induce cytotoxicity. The method for inducing cellular inflammation and pyroptosis was performed according to the intervention protocol for mouse epithelial cells described in Ref. [30]. Briefly, cells were seeded at a density of 1 × 10^6^ cells/mL in 6-well plates and cultured for 24 h. Then, the cells were challenged with 1 μg/mL lipopolysaccharide (LPS) for 5 h, followed by exposure with 5 mM adenosine triphosphate (ATP) for 12 h to induce cytotoxicity. For disulfiram intervention, 8 μM disulfiram was added 2 h prior to LPS stimulation [31,32].

### 2.19 Cell transfection

A lentiviral transfection system was employed to knockdown or overexpress MXRA7 in the DC-2 cell line. Short hairpin RNAs targeting MXRA7 (shRNA^MXRA7^) and negative control short hairpin RNAs (shRNA^NC^) were designed, constructed, and integrated into the MISSION TRC2 pLKO.5-puro vector (Sigma-Aldrich (Shanghai), Shanghai, China). For overexpression experiments, the MXRA7-specific overexpression vector (EV-MXRA7) and the empty vector control (EV) were integrated into the pCDH-CMV-MCS-EF1α-Puro vector (SBI, Mountain View, USA). All the above vector construction procedures were performed by Servicebio (Wuhan, China).

Subsequently, lentiviruses were packaged to generate small interfering RNAs (siRNAs) and mediate exogenous MXRA7 expression. The sequence of siRNA^NC^ was TTCTCCGAACGTGTCACGTTT, and that of siRNA^MXRA7^was CTGAAGAAGCCGAGGAAGATT; the coding sequence of MXRA7 used in this study was verified to be correct by sequencing. DC-2 cells were separately infected with lentiviruses carrying shRNA^MXRA7^, shRNA^NC^, the MXRA7 overexpression vector, or the empty vector. The culture medium was replaced after overnight incubation, and the cells were then transferred to a selection medium containing 2 μg/mL puromycin (Sigma-Aldrich) for screening. The cells were maintained and passaged in the selection medium until subsequent experimental assays or cryopreservation, ensuring that stable MXRA7 knockdown or overexpression was achieved at all experimental time points.

### 2.20 RNA extraction and qRT-PCR analysis

Total RNA was isolated from murine epididymal tissues or DC-2 and PC-1 cells following the standard TRIzol extraction protocol. Using 1 μg of total RNA as the template, complementary DNA (cDNA) was synthesized with the PrimeScript RT Reagent Kit (containing gDNA Eraser, Takara Bio Inc., Kusatsu, Japan) to eliminate genomic DNA contamination. Quantitative real-time polymerase chain reaction (qRT-PCR) assays were performed on the StepOnePlus Real-Time PCR System (Applied Biosystems, Foster City, USA), with SYBR Green Premix Pro Taq HS (Accurate Biotechnology, Changsha, China) used as the fluorescent dye.

Primers for MXRA7, IL-6, IL-1β, TNF-α, and the reference gene GAPDH were designed based on the corresponding gene sequences from the NCBI database and synthesized by Servicebio (Wuhan, China). The 20 μL reaction system consisted of the following components: 10 μL SYBR Green Premix, 0.4 μL forward primer (10 μM), 0.4 μL reverse primer (10 μM), 2 μL cDNA template, and 7.2 μL nuclease-free water. The amplification conditions were set as follows: initial denaturation at 95 °C for 30 s, followed by 40 cycles of denaturation at 95 °C for 5 s and annealing/extension at 60 °C for 30 s. After amplification, a melting curve analysis (65–95 °C) was performed to verify the specificity of the PCR products. The relative mRNA expression levels of target genes were calculated using the 2^−ΔΔCt^ method and normalized against the reference gene GAPDH. The primer sequences were as follows:MXRA7 (NM_026280.3): Forward 5′-GGGCCACCCACTGAAGAAC-3′,Reverse 5′-GTCTGACATCTCGCCAAAGGT-3′; IL-6 (NM_031168.2): Forward 5′-CAAGACTTCCATCAGTGC-3′,Reverse 5′-GGCAGTCTCCTCATTGAATCC-3′; IL-1β (NM_008361.4): Forward 5′-TGGCAATGAGGATGACTTTCTTAC-3′,Reverse 5′-GCTTGTGCTCTGCTGTGTGGT-3′; TNF-α (NM_013693.3): Forward 5′-CCTGTAGCCCACGTCTAGC-3′,Reverse 5′-GGTTTGCTACAACATGGGCTACA-3′.

### 2.21 Western blot (WB)

Epididymal tissues or cell pellets were homogenized by grinding in a lysis buffer containing RIPA buffer, PMSF protease inhibitor, and phosphatase inhibitors. After incubation on ice, the homogenates were centrifuged at 11,000 r·min^−1^ for 15 min at 4 °C (centrifugal radius: 10 cm). The supernatant was collected to extract nuclear proteins and total tissue proteins. Protein concentrations were determined using the bicinchoninic acid (BCA) assay (Beyotime Biotechnology, Shanghai, China). Subsequently, the protein samples were boiled at 100 °C for 10 min, followed by sodium dodecyl sulfate-polyacrylamide gel electrophoresis (SDS-PAGE) procedures including gel preparation, sample loading, and electrophoresis. The separated proteins were transferred onto polyvinylidene fluoride (PVDF) membranes. The membranes were blocked with 5% non-fat milk on a shaking platform at room temperature for 1.5 h, then washed three times with TBST. Primary antibody working solutions were added, and the membranes were incubated overnight at 4 °C. The primary antibodies were recovered, and the membranes were washed three times with TBST again. HRP-conjugated secondary antibodies (1:10,000 dilution) were added and incubated for 1 h on a shaker. After washing three times with TBST, chemiluminescent substrate was added, and exposure and development were performed using an SCG-W3000 PLUS chemiluminescence imaging system (Servicebio, Wuhan, China). The protein bands were quantified using Image J software.

### 2.22 LDH release assay

Cells were seeded in 96-well culture plates and subjected to the corresponding treatments. After treatment, all cell culture supernatants were collected, centrifuged at 400 × g for 5 min, and analyzed using an LDH cytotoxicity assay kit (Beyotime Biotechnology, Shanghai, China). Specifically, 120 μL of supernatant from each well was transferred to a new 96-well plate, followed by the addition of 60 μL of LDH detection reagent. The mixture was incubated at room temperature in the dark for 30 min. The absorbance was then measured at 490 nm. The relative LDH release was calculated as follows: LDH release (%) = (sample LDH release − spontaneous LDH release)/(maximum LDH release − spontaneous LDH release) × 100. Spontaneous LDH release was assessed using untreated control cells, while maximum LDH release was evaluated by treating untreated cells with the LDH release reagent included in the kit [33].

### 2.23 Luciferase reporter assay

The firefly luciferase reporter plasmid pNFκB-luc and the reference plasmid pRL-TK (Beyotime Biotechnology, Shanghai, China) were co-transfected into cells using Lipofect5000 Prime DNA/siRNA Transfection Reagent (Biodai Biotechnology, Changzhou, China). At 48 h post-transfection, luciferase activity was measured in accordance with the manufacturer's instructions for the Dual-Luciferase Reporter Assay Kit (Beyotime Biotechnology, Shanghai, China). The transcriptional activity of NF-κB was calculated as the ratio of firefly luciferase activity to Renilla luciferase activity [34].

### 2.24 Phos-tag SDS-PAGE

Total cellular and tissue proteins were extracted and subjected to electrophoresis on Phos-tag SDS-PAGE precast gels (Abmart, Shanghai, China) to separate phosphorylated and non-phosphorylated proteins. After sample loading and electrophoresis, the gels were first washed twice with 5 mM EDTA in methanol-free transfer buffer for 10 min each, followed by a single wash with methanol-free transfer buffer alone [35]. Membrane transfer was then performed, and the remaining steps were identical to those for Western blotting.

### 2.25 Co-immunoprecipitation (Co-IP)

Cells were lysed in ice-cold RIPA lysis buffer supplemented with protease and phosphatase inhibitors (Thermo Fisher Scientific, Waltham, USA) for 30 min. After centrifugation at 12,000 × g for 15 min at 4 °C, the supernatant was collected and incubated with 20 μL of protein A/G agarose beads (Santa Cruz Biotechnology, Dallas, USA) for 1 h at 4 °C with gentle shaking for pre-clearing. Subsequently, 2–5 μg of primary antibody or normal IgG (used as a negative control) was added to the pre-cleared lysate and incubated overnight at 4 °C, followed by the addition of 30 μL of protein A/G agarose beads and further incubation for 4 h at 4 °C. The beads were washed five times with cold PBS containing 0.1% Triton X-100. The immunoprecipitated complexes were resuspended in 2× SDS-PAGE loading buffer, and the target proteins were eluted by boiling for 10 min. Finally, the interacting proteins were detected via WB assay using specific antibodies.

### 2.26 In vitro phosphorylation assay

The phosphorylation assay in vitro was performed as described previously [35] with minor modifications. The MXRA7-containing complexes immunoprecipitated with MXRA7 antibody were washed twice with lysis buffer and once with wash buffer (40 mM HEPES pH 7.6, 10 mM MgCl_2_, 1 mM EGTA, 1 mM DTT). The complexes were then incubated with recombinant active PKCα in kinase buffer (40 mM HEPES pH 7.6, 10 mM MgCl_2_, 1 mM EGTA, 1 mM DTT, 2.5 mM β-glycerophosphate, 0.01% Triton X-100, 200 μM ATP) in the presence or absence of ATP at 30 °C for 30 min. The reaction was terminated by adding 5× Laemmli sample buffer, followed by heating at 95 °C for 5 min. Subsequently, the samples were subjected to Phos-tag SDS-PAGE and immunoblotted with MXRA7 antibody to detect the phosphorylation level of MXRA7.

### 2.27 Sperm in vitro incubation

DC-2 cells with MXRA7 knockdown or overexpression and treated with ATP + LPS were washed and incubated in IMDM medium (without fetal bovine serum (FBS) and penicillin/streptomycin) for 24 h. The cells were removed and the conditioned medium was retained. Sperm were collected from the cauda epididymidis of normal ICR mice and incubated at 37 °C for 30 min. The obtained samples were centrifuged at 400 g for 5 min at room temperature to separate mouse epididymal sperm from epididymal fluid. The prepared sperm samples were co-incubated with the prepared conditioned medium for 12 h at room temperature in a 5% CO_2_ atmosphere, and then processed and analyzed according to the aforementioned sperm treatment methods. For experiments involving exogenous recombinant MXRA7 intervention, mouse rMXRA7 (Cusabio, Wuhan, China; derived from *E. coli*) was first depleted of endotoxin using Pierce High Capacity Endotoxin Removal Spin Columns (Thermo Fisher Scientific, Waltham, USA). The protein was then incubated with normal sperm samples at concentrations of 0.1, 1, 10, and 50 μg/mL for 12 h at room temperature under 5% CO_2_ to assess sperm parameters and determine the safe dose. Subsequently, conditioned medium was prepared as described above (DC-2 cells treated with ATP + LPS were washed, replenished with fresh medium, incubated for 24 h, and the cell-free supernatant was retained). Normal sperm samples were co-incubated with this conditioned medium together with the maximum safe dose of rMXRA7 for 12 h at room temperature under 5% CO_2_, after which sperm parameters and supernatant inflammatory cytokine levels were measured. Heat-inactivated rMXRA7 (HI-rMXRA7) and the recombinant protein vehicle (Veh) served as negative controls. HI-rMXRA7 was prepared by heating rMXRA7 at 95°C for 10 min in a thermomixer, immediately cooling on ice for 2 min, and briefly centrifuging to collect the condensate.

### 2.28 Statistical analysis

Data are presented as mean ± standard deviation (SD). Selected images were analyzed using ImageJ software. Statistical differences between two groups were determined by the student t-test, while differences among multiple groups were assessed by one-way analysis of variance (ANOVA), followed by Tukey's post-hoc test for multiple comparisons. A p-value <0.05 was considered statistically significant.

## 3 Result

### 3.1 EIF-induced oxidative stress: reproductive inflammation, sperm damage and MXRA7 association

**Fig. 1:**
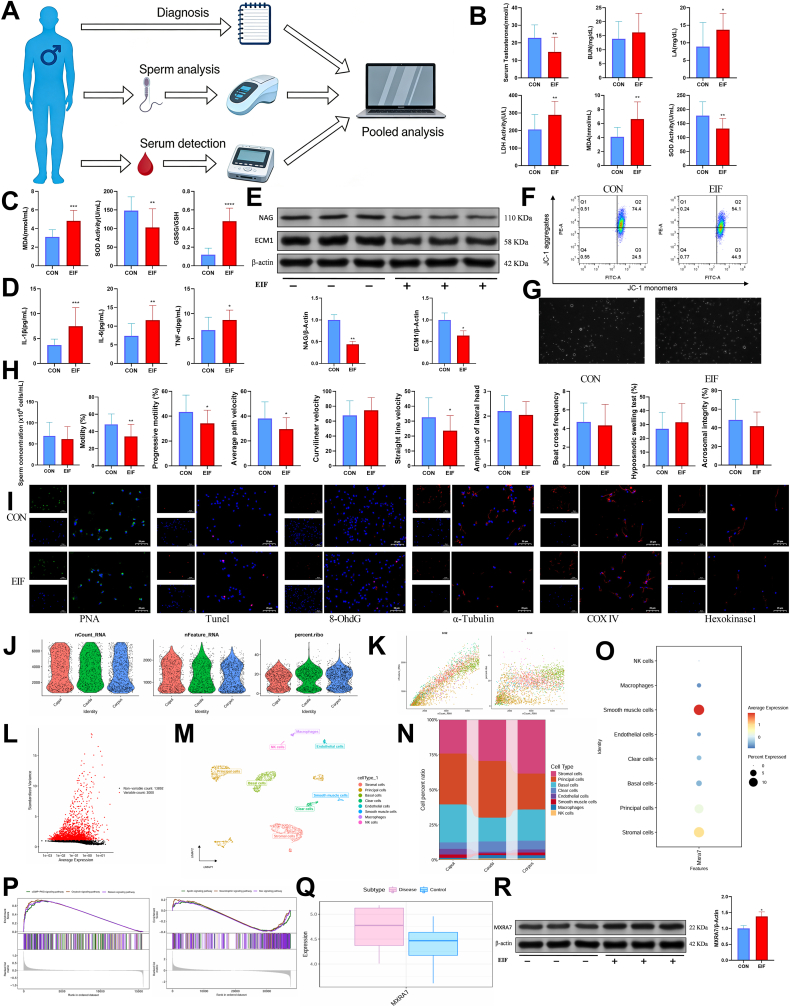
The reproductive system of EIF patients exhibits oxidative stress and inflammation, accompanied by significant upregulation of MXRA7 Note: A: Study design for human subjects; B: Serum biomarker levels in human subjects; C: Oxidative stress markers in seminal plasma; D: Inflammatory cytokine levels in seminal plasma; E: WB analysis of NAG and ECM1 levels in seminal plasma; F: JC-1 flow cytometry analysis of sperm mitochondrial membrane potential; G-H: Sperm quality parameters, acrosome integrity and hypoosmotic swelling test; I: Immunofluorescence staining of sperm smears: target proteins were stained red, and nuclei were counterstained blue with DAPI, Scale bar = 50 μm; J-K: Quality control of single-cell RNA sequencing data; L: Identification of 3000 highly variable genes; M: Annotation of 8 epididymal cell clusters identified by UMAP dimensionality reduction; N: Bar plot showing the proportions of different epididymal cell types across epididymal segments; O: Expression pattern of MXRA7 in various epididymal cell types; P: GSEA of MXRA7 (left: GSE145443, right: GSE199903); Q: Differential expression of MXRA7 between normal and epididymitis samples; R: Western blotting analysis of MXRA7 levels in semen. ∗p < 0.05, ∗∗p < 0.01, ∗∗∗p < 0.001.

To investigate the effects of EIF on male fertility, sperm parameters were measured in EIF patients and compared with those in healthy controls (Fig. 1A). Serum biomarkers in EIF patients (Fig. 1B) indicated significant reductions in testosterone levels, metabolic waste clearance, and antioxidant capacity. Analysis of seminal plasma samples from EIF patients revealed elevated oxidative stress and inflammatory levels (Fig. 1C–D). Decreased levels of N-acetyl-β-glucosaminidase (NAG) and extracellular matrix protein 1 (ECM1), two epididymal function-related proteins [36,37], suggested impaired epididymal function (Fig. 1E). Furthermore, results from JC-1 flow cytometry directly demonstrated impaired sperm mitochondrial membrane potential in EIF patients [26] (Fig. 1F), further confirming that spermatozoa were subjected to oxidative stress and subsequent inflammatory damage. Comprehensive assessment of sperm quality parameters (Fig. 1H) showed that EIF patients exhibited significant impairments in sperm motility, straight-line velocity (VSL), and average path velocity (VAP). Among them, the EIF-H patients exhibited significant decreases in sperm concentration and acrosome integrity in addition to impaired motility (Fig. S1C). Immunofluorescence staining of spermatozoa (Fig. 1I) was performed to evaluate acrosome integrity (PNA), DNA damage (TUNEL, 8-OHdG), and motility-related proteins (α-tubulin, COX IV, Hexokinase I). Sperm from EIF patients showed mild, non-significant acrosome and DNA damage, whereas staining intensity of motility-related proteins was significantly decreased. These findings collectively indicate that EIF exerts detrimental effects on sperm motility.

MXRA7 may be associated with epididymal inflammation, and we intended to further investigate this through bioinformatics analysis. Given the lack of an EIF-specific epididymitis-related dataset, we performed transcriptomic analysis of the epididymitis-related datasets (GSE145443, GSE199903) from the GEO database, with the aim of characterizing the cellular expression profile of MXRA7 in normal epididymal tissue and preliminarily exploring the expression trends of MXRA7 in epididymal tissue under general inflammatory conditions. After quality control, cells with outlier values or fewer than 500 detected genes were filtered out, followed by doublet removal. A total of 2611 cells were retained for subsequent analysis. Violin plots and scatter plots were generated using the filtered data (Fig. 1J–K), and 3000 highly variable genes were identified (Fig. 1L). The data were then subjected to normalization, scaling, PCA, and Harmony batch correction. Dimensionality reduction using UMAP identified 12 cell clusters, which were annotated into 8 cell types based on canonical markers: stromal cells, principal cells, basal cells, clear cells, endothelial cells, smooth muscle cells, macrophages, and NK cells (Fig. 1M). The proportions of these 8 cell types across different epididymal segments were visualized using bar plots (Fig. 1N). Subsequently, the DotPlot and FeaturePlot functions in the Seurat R package were used to visualize Mxra7 expression at the single-cell level (Fig. 1O), and differential expression analysis of MXRA7 between normal and epididymitis samples was conducted (Fig. 1Q). MXRA7 was most highly expressed in smooth muscle cells, principal cells, and clear cells, which constitute the major cell types of the epididymal epithelium. Furthermore, MXRA7 expression was significantly upregulated in general epididymal inflammatory samples. GSEA of the GSE145443 and GSE199903 datasets (Fig. 1P) revealed that MXRA7 was enriched in multiple signaling pathways, including the cGMP-PKG signaling pathway, oxytocin signaling pathway, relaxin signaling pathway, apelin signaling pathway, neurotrophin signaling pathway, and Ras signaling pathway. Finally, direct measurement of MXRA7 levels in human seminal plasma demonstrated significantly elevated MXRA7 concentrations in EIF patients, indicating that MXRA7 may be involved in the pathogenesis of EIF-induced sperm damage.

### 3.2 EIF induces oxidative stress in the mouse reproductive system and impairs sperm quality

**Fig. 2:**
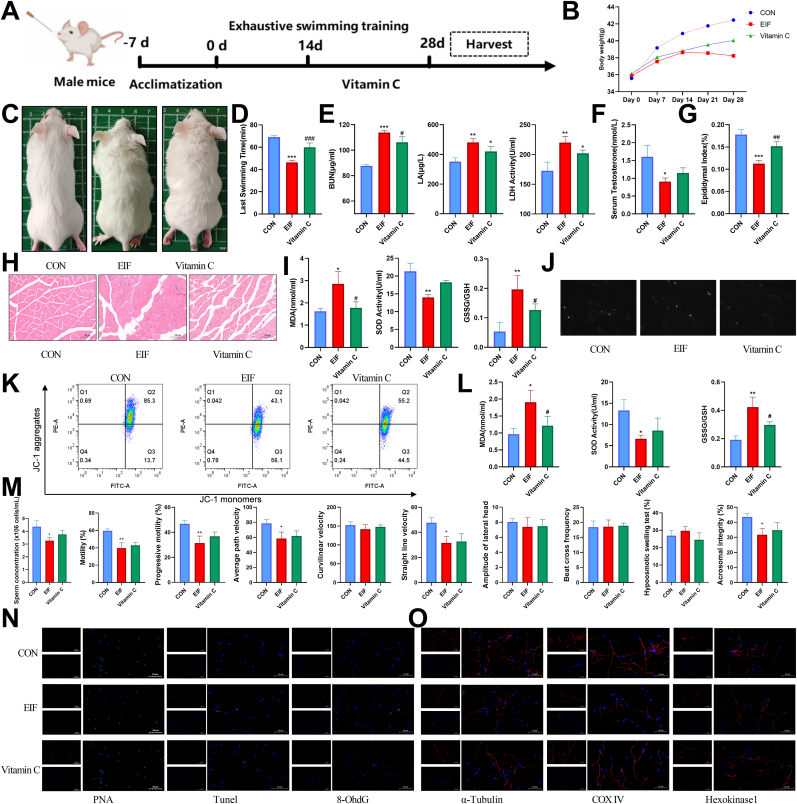
Oxidative stress and impaired sperm quality in the reproductive system of EIF mice Note: A: Experimental timeline for animal model establishment and intervention; B: Body weight changes of mice; C: Representative photographs of mouse morphology; D: Last exhaustive swimming time of mice; E: Serum fatigue markers (LA, BUN, LDH); F: Serum testosterone levels; G: Epididymal index (epididymal weight/total body weight); H: HE staining of gastrocnemius muscle; I: Serum oxidative stress markers; J: Morphological observation of mouse sperm; K: JC-1 flow cytometry analysis of sperm mitochondrial membrane potential; L: Oxidative stress markers in epididymal fluid; M: Sperm quality parameters, acrosome integrity and hypoosmotic swelling test; N: Immunofluorescence staining of sperm PNA (acrosome marker) and DNA damage markers; red fluorescence indicates target proteins, blue fluorescence indicates DAPI-stained nuclei, Scale bar = 50 μm; O: Immunofluorescence staining of sperm motility-related proteins, Scale bar = 50 μm ∗P < 0.05, ∗∗P < 0.01, ∗∗∗P < 0.001 vs. CON group; #P < 0.05, ##P < 0.01, ###P < 0.001 vs. EIF group.

To further investigate the effects of EIF on sperm function, we established an EIF mouse model (Fig. 2A) and included a vitamin C intervention group to examine whether this conventional antioxidant and fatigue supplement could ameliorate sperm-related impairments. After the intervention period, EIF model mice exhibited significant reductions in body weight (Fig. 2B), fur luster and smoothness (Fig. 2C), and exercise capacity (Fig. 2D). Serum analysis of metabolic parameters (LA, BUN, LDH) (Fig. 2E) and testosterone levels (Fig. 2F) revealed markedly elevated metabolic waste products and decreased testosterone concentrations in EIF mice. Histomorphological observation (Fig. 2H) showed obvious edema in the gastrocnemius muscle of EIF mice with widened inter-myofibrillar spaces, indicative of muscle fatigue. Detection of serum oxidative stress markers (MDA, SOD, GSSG/GSH) (Fig. 2I) demonstrated increased systemic oxidative stress in EIF mice, which was partially alleviated by vitamin C intervention. Collectively, these results confirmed the successful establishment of the mouse EIF model and the moderate anti-fatigue effect of vitamin C.

To evaluate the impact of EIF on the reproductive system, we found that the epididymal index of EIF mice was significantly decreased (Fig. 2G), which was partially restored after vitamin C treatment. JC-1 flow cytometry analysis of mouse sperm (Fig. 2K) revealed a significant reduction in sperm mitochondrial membrane potential. Meanwhile, measurement of oxidative stress markers in epididymal fluid (Fig. 2L) indicated the presence of oxidative stress in the reproductive system and the epididymal microenvironment of EIF mice, which was ameliorated by vitamin C.

Changes in sperm quality (Fig. 2M) were similar to those observed in human EIF-H patients (Fig. S1C), characterized primarily by a marked decline in sperm motility, accompanied by significant reductions in sperm concentration and acrosome integrity. This may be attributable to the fact that the weight-loaded swimming training in mice approaches the physical limit more closely, and therefore better resembles the condition of EIF-H patients with more severe fatigue. Immunofluorescence staining of sperm smears showed a slight decrease in acrosome integrity, while no statistically significant changes in DNA damage were detected (Fig. 2N). Proteins associated with sperm motility (Fig. 2O) also exhibited obvious impairment, similar to the findings in humans.

Notably, vitamin C did not exert a significant effect on overall sperm quality in EIF model mice. Although improvements were observed in the epididymal index and immunofluorescence intensity of sperm motility-related proteins, all conventional sperm parameters remained statistically non-significant. Nevertheless, this does not negate the beneficial effects of vitamin C on other EIF-related pathologies, particularly the systemic oxidative stress involving the reproductive system. Taken together, these data demonstrate that EIF induces oxidative stress in the reproductive system and impairs sperm quality, especially sperm motility.

### 3.3 EIF induces pyroptosis and significant MXRA7 expression in mouse epididymal tissue

**Fig. 3:**
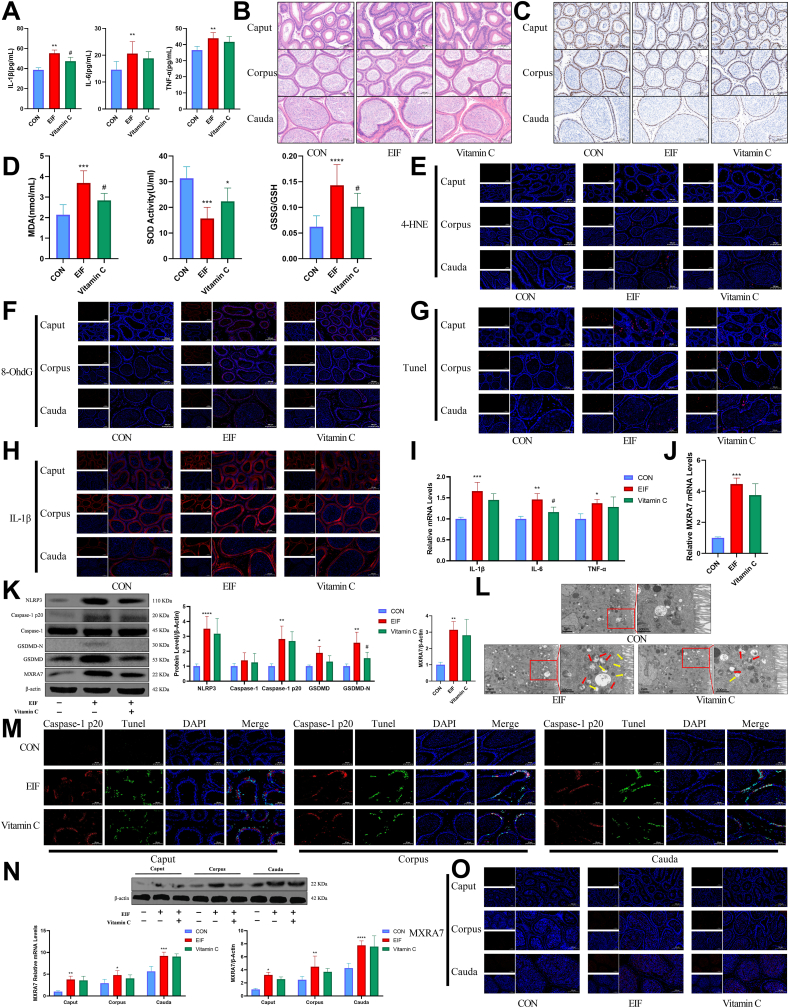
Epididymal damage and MXRA7 expression profiles in EIF mice Note: A: Inflammatory factor levels in mouse epididymal fluid; B: HE staining showing the morphology of different epididymal regions in each group; C: NAG immunohistochemical staining showing the functional status of different epididymal regions; D: Antioxidant indicators in epididymal tissues of each group; E: Immunofluorescence staining of 4-HNE in different epididymal segments, Scale bar = 100 μm; F: Immunofluorescence staining of 8-OhdG in different epididymal segments, Scale bar = 100 μm; G: TUNEL staining showing apoptosis in different epididymal regions, Scale bar = 100 μm; H: Immunofluorescence staining of IL-1β in different epididymal segments, Scale bar = 100 μm; I-J: mRNA expression levels of inflammatory factors and MXRA7 in mouse epididymis; K: Western blot analysis of pyroptosis-related proteins and MXRA7 protein expression in the epididymis; L: TEM observation of epididymal epithelial cell ultrastructure in mouse. Scale bars = 2 μm and 500 nm. Red arrows: mitochondrial swelling and damage; yellow arrows: abnormal cellular perforations; M: Immunofluorescence double staining of Caspase-1 p20 (red) and TUNEL (green) in epididymal tissue. TUNEL and Caspase-1 p20 double-positive cells indicate pyroptosis. Scale bar = 50 μm; N: Protein and mRNA expression levels of MXRA7 in different segments of the epididymis of mice in each group; O: Immunofluorescence staining of MXRA7 in different epididymal segments, Scale bar = 100 μm ∗P < 0.05, ∗∗P < 0.01, ∗∗∗P < 0.001, ∗∗∗∗P < 0.0001 vs. CON group; #P < 0.05, ##P < 0.01, ###P < 0.001, ####P < 0.0001 vs. EIF group.

Based on the above findings that oxidative stress was significantly elevated in the epididymal fluid of EIF mice (Fig. 2L), combined with the decreased epididymal functional marker proteins and increased inflammatory factors in the seminal plasma of human EIF patients (Fig. 1D–E), we hypothesized that EIF may induce epididymal inflammation. Subsequent detection of significantly increased inflammatory factor levels in the epididymal fluid of EIF mice (Fig. 3A) further supported this hypothesis. To further characterize the epididymal inflammation, we investigated three distinct anatomical regions of the epididymis: the caput, corpus, and cauda. Histological observation (Fig. 3B) showed that the epididymal walls were significantly thickened in all three regions of EIF mice, with the most prominent changes in the caput, indicating active inflammation. Neutral α-glucosidase (NAG) is a functional epididymal marker that is almost exclusively derived from the epididymis and reliably reflects epididymal secretory function [36]. Immunohistochemical staining for NAG (Fig. 3C) confirmed impaired epididymal function in EIF mice.

Detection of antioxidant indicators in epididymal tissues (Fig. 3D) demonstrated that oxidative stress occurred locally in the epididymis of EIF mice, which was partially attenuated by vitamin C intervention. Immunofluorescence staining showed increased accumulation of 4-HNE and 8-OhdG in the epididymal tissue of EIF mice (Fig. 3E–F), with the highest expression levels observed in the caput region. Vitamin C treatment significantly reduced their expression, confirming that EIF induces oxidative stress-mediated epididymal damage and that vitamin C exerts an ameliorative effect.

TUNEL staining (Fig. 3G) indicated significant apoptosis in the caput and corpus regions of EIF mice. Immunofluorescence staining for IL-1β and qPCR analysis of inflammatory factors (Fig. 3H–I) confirmed a robust inflammatory response in the epididymal tissue of EIF mice, which may be triggered by oxidative stress injury. Further WB analysis and TEM observations of epididymal epithelial cells (Fig. 3K–L) demonstrated that the epididymis of EIF mice not only exhibited oxidative stress and inflammation, but also underwent pyroptosis. TUNEL and Caspase-1 p20 double immunofluorescence staining (Fig. 3M) revealed that the number of Caspase-1 p20/TUNEL double-positive cells was significantly higher than that of TUNEL single-positive cells in the epididymal tissue of EIF mice, suggesting that pyroptosis may be the dominant mode of cell death. Collectively, these results confirmed that EIF induces epididymal inflammation and pyroptosis, which can be partially ameliorated by vitamin C.

Detection of MXRA7 expression in epididymal tissues revealed that it was significantly upregulated in the epididymis of EIF mice (Fig. 3J–K), and its expression levels also differed significantly among different epididymal segments (Fig. 3N–O). Multiple experimental approaches consistently showed that MXRA7 expression was significantly higher in the cauda than in the caput, suggesting that MXRA7 may play an important role in the pathogenesis of EIF-induced epididymal inflammation.

### 3.4 MXRA7 expression is negatively correlated with the severity of inflammation in different epididymal segments

**Fig. 4:**
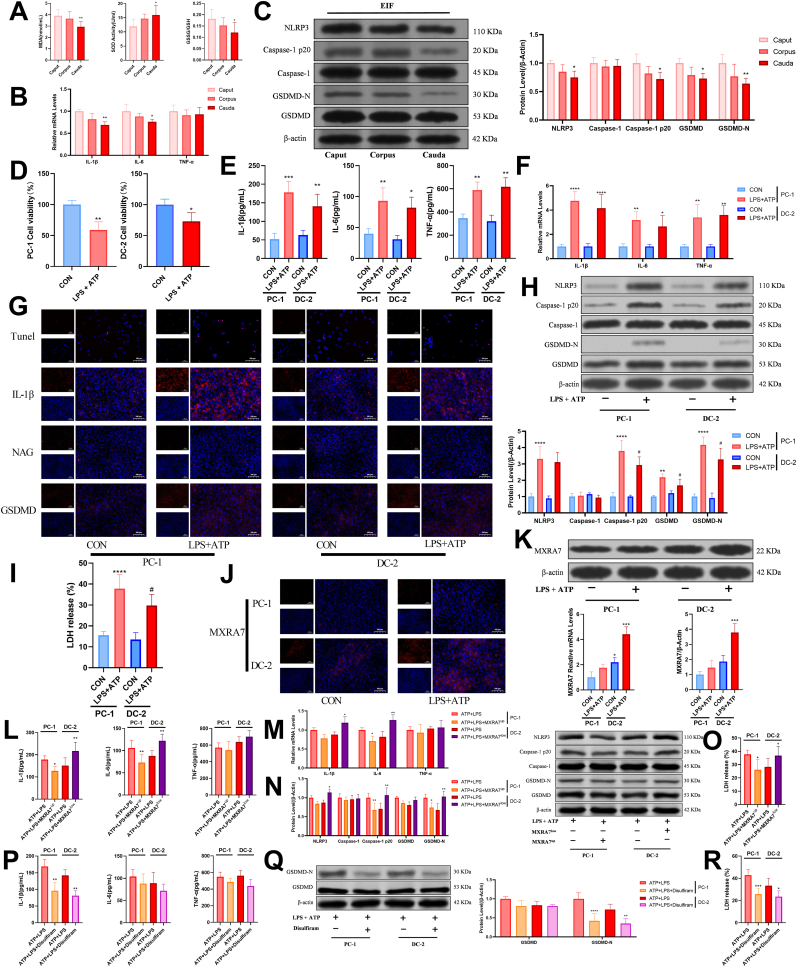
Segmental differences in MXRA7 expression and inflammation in EIF mouse epididymis and epididymal cell lines Note: A: Antioxidant indicators in different epididymal segments of EIF mice; B: mRNA expression levels of inflammatory factors in different epididymal segments of EIF mice; C: Pyroptosis levels in different epididymal segments of EIF mice; D: Cell viability detected by CCK-8 assay; E-F: Inflammatory factor levels in cell supernatants and cellular mRNA expression levels; G: Immunofluorescence staining of PC-1 and DC-2 cells, Scale bar = 100 μm; H: Western blot analysis and quantification of pyroptosis-related proteins; I: LDH release assay; J-K: WB, qPCR and immunofluorescence for MXRA7 expression, Scale bar = 100 μm; L-M: Inflammatory cytokines in supernatants and cellular mRNA levels; N: WB analysis of pyroptosis-related proteins and quantification; O: LDH release assay; P: Levels of inflammatory cytokines in the cell supernatant under disulfiram intervention; Q: Western blot detection of pyroptosis-related proteins and their quantification under disulfiram intervention; R: Detection of pyroptosis by LDH release assay under disulfiram intervention. ∗P < 0.05, ∗∗P < 0.01, ∗∗∗P < 0.001, ∗∗∗∗P < 0.0001 vs. caput group or vs. control group of the same cell line; #P < 0.05, ##P < 0.01, ###P < 0.001, ####P < 0.0001 vs. the same indicator in the other cell line.

To further validate the differences and potential associations between pyroptosis and MXRA7 expression across different epididymal segments, we performed detailed analyses on the caput, corpus, and cauda epididymidis of EIF mice. We found that the cauda epididymidis exhibited significantly lower levels of oxidative stress (Fig. 4A), inflammatory factors (Fig. 4B), and pyroptosis (Fig. 4C) compared to the caput. The severity of EIF-induced epididymal inflammation gradually decreased from the caput to the cauda. In contrast, MXRA7 expression in EIF mouse epididymal tissue gradually increased from the caput to the cauda (Fig. 3L–N), showing a negative correlation with inflammation severity. This further supports the existence of a functional link between the two, suggesting that MXRA7 may exert an anti-inflammatory effect.

To eliminate potential confounding factors such as anatomical and structural differences in vivo, we used the caput-derived PC-1 cell line and cauda-derived DC-2 cell line for in vitro experiments. Pyroptosis was induced in both cell lines using the same dose of ATP and LPS (the H_2_O_2_-induced cell model exhibited similar oxidative stress levels to the ATP + LPS-induced model but was less potent in upregulating MXRA7, suggesting that MXRA7 exerts its effects primarily through inflammatory pathways) (Fig. S2A–B). We then compared pyroptosis levels and MXRA7 expression between the two cell lines. In terms of cell viability (Fig. 4D), the PC-1 pyroptosis model showed a more significant statistical difference compared to the control group. For inflammatory factors (Fig. 4E–F), PC-1 cells exhibited slightly more significant differences in IL-1β and IL-6 levels, while no significant difference was observed for TNF-α. Immunofluorescence staining (Fig. 4G) showed that ATP/LPS-induced PC-1 cells had slightly higher fluorescence intensity for IL-1β and TUNEL staining, slightly lower NAG fluorescence intensity compared to DC-2 cells, and no significant difference in GSDMD immunofluorescence. WB analysis (Fig. 4H) and LDH release assay (Fig. 4I) showed that DC-2 cells exhibited lower levels of apoptosis and pyroptosis (Caspase-1 p20 and GSDMD-N) compared with PC-1 cells, with significant statistical differences. Detection of MXRA7 levels (Fig. 4J–K) revealed that the protein and mRNA levels of MXRA7 were significantly increased in DC-2 cells compared with PC-1 cells under the same dose of LPS and ATP induction. Furthermore, gradient doses of vitamin C [[38], [39], [40]] had no effect on MXRA7 expression in normal DC-2 cells (Fig. S4A), whereas in ATP- and LPS-induced DC-2 cells, intervention with gradient doses of vitamin C (Fig. S4A–C) resulted in a partial reduction of MXRA7 levels as the severity of inflammation and pyroptosis decreased, suggesting that vitamin C does not exert a direct effect on MXRA7.

**Fig. 5:**
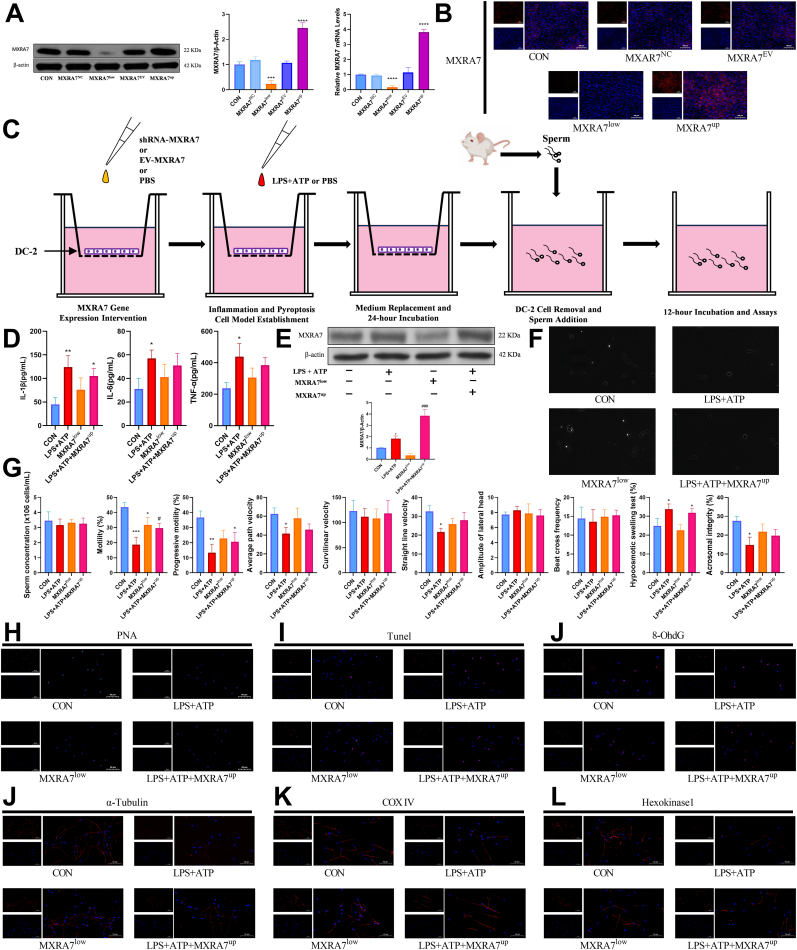
MXRA7 alleviates pyroptosis-induced sperm damage in DC-2 cells Note: A: MXRA7 protein and mRNA expression levels; B: Immunofluorescence staining of MXRA7 in DC-2 cells, Scale bar = 100 μm; C: Schematic diagram of the in vitro sperm co-incubation procedure; D: Inflammatory factor levels in the supernatant after incubation; E: Western blot detection of MXRA7 expression levels in the culture medium; F-G: Sperm quality analysis by automatic sperm analyzer; H-L: Immunofluorescence detection of acrosome integrity, DNA damage and motility-related indicators in incubated sperm, Scale bar = 50 μm ∗P < 0.05, ∗∗P < 0.01, ∗∗∗P < 0.001, ∗∗∗∗P < 0.0001 vs. CON group; #P < 0.05, ##P < 0.01, ###P < 0.001, ####P < 0.0001 vs. LPS + ATP group.

To rule out the possible inherent immune differences between the two cell lines other than MXRA7, MXRA7 was overexpressed in the PC-1 cell line using a lentiviral infection system (Fig. S3), and MXRA7 was knocked down in the DC-2 cell line (Fig. 5A–B) to further examine the effect of MXRA7 changes on inflammatory responses in the same cell line background. The results showed that PC-1 cells overexpressing MXRA7 had lower levels of inflammation and pyroptosis, while DC-2 cells with MXRA7 knockdown had higher levels of inflammation and pyroptosis (Fig. 4L–O), directly indicating that MXRA7 exerts an anti-inflammatory effect.

To clarify the mode of cell death, disulfiram was added prior to ATP and LPS intervention (Fig. 4P–R), further confirming that pyroptosis is the dominant mode of cell death under ATP and LPS induction.

The above results demonstrate that DC-2 cell line exhibited lower levels of pyroptosis and inflammation than PC-1 cell line under the stimulation of equivalent doses of ATP and LPS, indicating that the cauda epididymidis possesses stronger anti-inflammatory capacity than the caput epididymidis. Further experiments investigating the differential expression of MXRA7 between these two segments and its anti-inflammatory effect revealed that this may be attributed to the fact that high expression of MXRA7 in DC-2 cells attenuated the inflammatory response to a certain extent. The underlying mechanisms warrant further investigation and validation.

### 3.5 MXRA7 in cauda epididymal epithelial cells attenuates pyroptosis-induced sperm damage

Based on the potential association between epididymal inflammation and MXRA7, combined with the characteristic that MXRA7 is a secretory protein, we further verified the direct effect of epididymal MXRA7 expression on sperm under epididymitis conditions. We used the DC-2 cell line (cauda epididymal epithelial cell line) with high endogenous MXRA7 expression as the primary research object, and knocked down or overexpressed MXRA7 in DC-2 cells via a lentiviral infection system to examine the specific biological role of MXRA7. The MXRA7 knockdown and overexpression models in DC-2 cells were successfully established and validated (Fig. 5A–B).

After inducing inflammation and pyroptosis in MXRA7-knockdown or MXRA7-overexpressing DC-2 cells, the medium was replaced and the cells were incubated for 24 h. The conditioned medium was collected and co-incubated with sperm from normal ICR mice to evaluate sperm function (Fig. 5C). After incubation, the supernatant was harvested to measure inflammatory factor levels (Fig. 5D), and the results showed that inflammatory factor production was partially inhibited by MXRA7. The MXRA7 protein level in the supernatant was also measured (Fig. 5E), which confirmed the presence of MXRA7 protein in the supernatant, and its level was positively correlated with MXRA7 expression in DC-2 cells, suggesting that increased MXRA7 expression in DC-2 cells leads to a corresponding increase in its protein secretion.

Sperm quality after co-incubation was analyzed using an automatic sperm analyzer (Fig. 5F–G). The treated sperm exhibited a similar phenotype to that of sperm from EIF mice in the aforementioned experiments, characterized by a significant reduction in sperm motility. MXRA7 overexpression significantly alleviated this decline in sperm motility, while MXRA7 knockdown markedly exacerbated the inhibition of sperm motility. Immunofluorescence results (Fig. 5H–L) also confirmed that the protective effect of MXRA7 was mainly reflected in the improvement of sperm motility-related indicators. Interestingly, MXRA7 also appeared to exert a moderate protective effect on acrosome integrity.

Taken together, sperm treated with conditioned medium from pyroptotic DC-2 cells recapitulated the sperm defects observed in EIF model mice, suggesting that inflammation and pyroptosis of epididymal epithelial cells may be one of the key mechanisms underlying EIF-induced sperm damage. MXRA7 indeed exerts a protective effect on sperm, particularly on sperm motility, which may be attributed to the ability of MXRA7 to enhance the anti-inflammatory capacity of epididymal epithelial cells.

To further investigate the protective effect of MXRA7 on spermatozoa, gradient doses of recombinant mouse MXRA7 protein (rMXRA7) were directly applied to spermatozoa from normal ICR mice in vitro (Fig. S5A). The results showed that, except for the excessively high dose (50 μg/mL), which markedly impaired sperm motility, some doses appeared to exert a slight improving effect on sperm motility, but no statistically significant differences were detected. The above experimental procedure was then repeated (Fig. 5C): conditioned medium was collected from DC-2 cells after the induction of inflammation and pyroptosis and incubated with spermatozoa, and the validated maximum safe dose of rMXRA7 (10 μg/mL) was added to the conditioned medium and co-incubated with normal spermatozoa. Sperm parameters and supernatant inflammatory factor levels were then measured (Fig. S5B–C). The results showed that, although a very mild increase in sperm motility and a decrease in inflammatory factors were observed after the addition of rMXRA7, these changes still did not reach statistical significance, suggesting that the indirect protection of spermatozoa by MXRA7, achieved through the alleviation of epididymal epithelial cell inflammation, may play a predominant role.

### 3.6 MXRA7 inhibits the NF-κB signaling pathway and attenuates inflammation

**Fig. 6:**
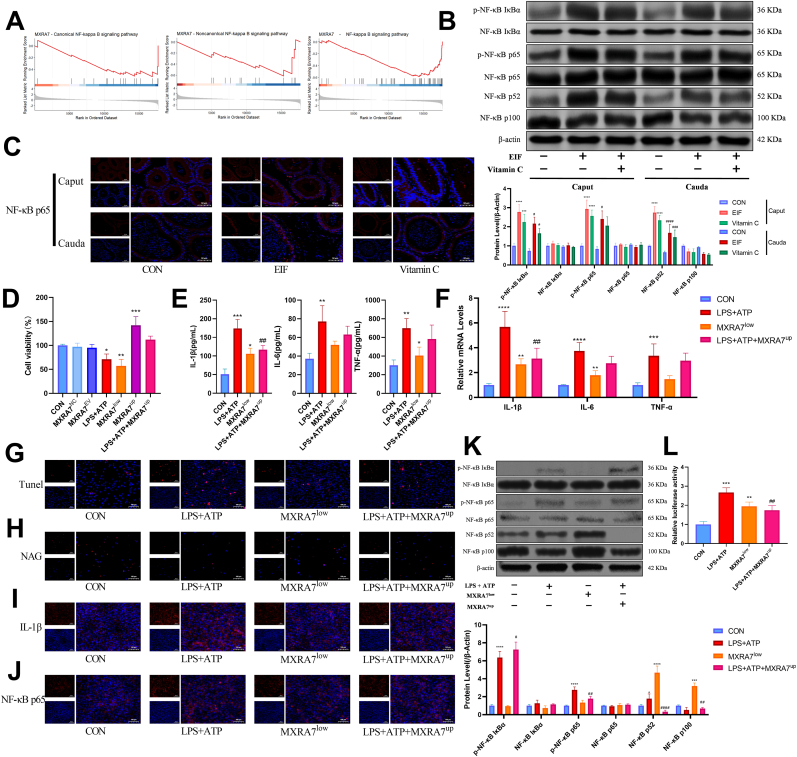
MXRA7 inhibits the activation of the NF-κB signaling pathway Note: A: GSEA analysis of the association between MXRA7 and canonical/non-canonical NF-κB pathways; B: Western blot analysis of NF-κB-related protein expression in the caput and cauda epididymidis of mice in each group; C: Immunofluorescence detection of p65 expression in mouse epididymal tissues of each group, Scale bar = 100 μm; D: DC-2 cell viability detected by CCK-8 assay; E-F: Inflammatory factor levels in supernatants and cellular inflammatory factor mRNA levels; G-J: Immunofluorescence staining of DC-2 cells, Scale bar = 100 μm; K: Western blot analysis of key proteins in the NF-κB signaling pathway in DC-2 cells; L: pNFκB-luc luciferase reporter assay; results are presented as the ratio of firefly luciferase activity to Renilla luciferase activity. ∗P < 0.05, ∗∗P < 0.01, ∗∗∗P < 0.001, ∗∗∗∗P < 0.0001 vs. CON group; #P < 0.05, ##P < 0.01, ###P < 0.001, ####P < 0.0001 vs. LPS + ATP group.

Consistent with our previous findings that MXRA7 exerts anti-inflammatory effects, further bioinformatic analysis and literature review revealed a potential association between MXRA7 and the NF-κB signaling pathway, which was confirmed by GSEA (Fig. 6A). Detection of NF-κB signaling pathway-related proteins in the caput and cauda epididymidis of mice in each group (Fig. 6B) demonstrated significant activation of NF-κB and increased nuclear translocation of p65 in the epididymal tissue of EIF mice. Notably, the degree of NF-κB activation was significantly lower in the cauda epididymidis than in the caput, particularly the levels of p52/p100, which represent the non-canonical NF-κB pathway, were markedly lower in the cauda.

Subsequent in vitro experiments were performed. Interestingly, detection of DC-2 cell viability after MXRA7 gene manipulation and ATP + LPS intervention (Fig. 6D) showed that MXRA7 knockdown severely impaired cell proliferation, even more severely than the pyroptosis model alone. In contrast, MXRA7 overexpression significantly enhanced cell viability, which was higher than that in the control group. These findings indicate that MXRA7 plays an important role in cell proliferation and differentiation, which is consistent with previous reports [21,41]. Detection of inflammatory factor levels in supernatants and cellular RNA (Fig. 6E–F) revealed that MXRA7 knockdown promoted inflammation, while MXRA7 overexpression attenuated ATP/LPS-induced inflammation. Meanwhile, MXRA7 knockdown induced cell apoptosis and increased IL-1β levels, while MXRA7 overexpression exerted the opposite effects. However, no significant effect on NAG expression was observed (Fig. 6G–I). Detection of the NF-κB signaling pathway (Fig. 6J–K), consistent with the in vivo experimental results, showed that MXRA7 inhibited the NF-κB signaling pathway, with moderate effects on p65 activation and nuclear translocation as well as p52/p100 levels. Notably, the inhibitory effect on p52/p100 was particularly pronounced, indicating that MXRA7 predominantly inhibits the non-canonical NF-κB pathway. Subsequent pNFκB-luc luciferase reporter assay (Fig. 6L) further confirmed that MXRA7 suppresses the NF-κB signaling pathway.

### 3.7 PKCα mediates the expression and phosphorylation of MXRA7

**Fig. 7:**
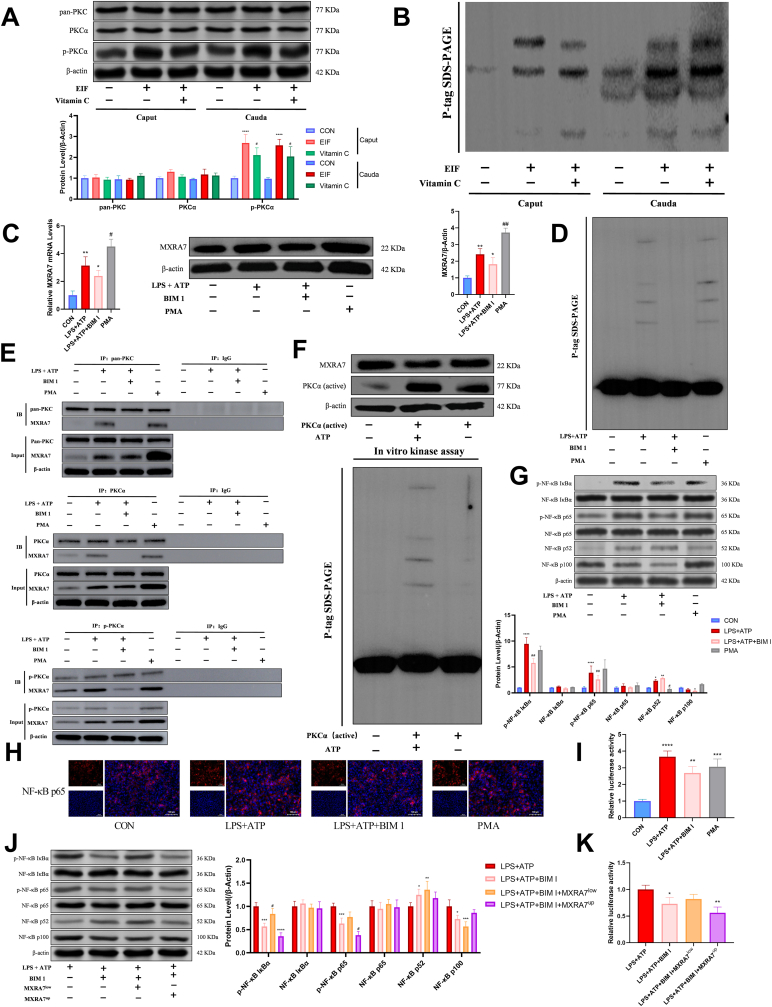
Activated PKCα phosphorylates MXRA7 Note: A: Western blot analysis of PKCα activation in mice of each group; B: Phos-tag SDS-PAGE separation of phosphorylated and non-phosphorylated MXRA7 proteins in mouse epididymal tissues of each group; C: MXRA7 protein and mRNA expression levels in DC-2 cells; D: Phos-tag SDS-PAGE separation of phosphorylated and non-phosphorylated MXRA7 proteins in DC-2 cells; E: Co-IP detection of the interactions between pan-PKC, PKCα, p-PKCα and MXRA7; F: In vitro phosphorylation assay of MXRA7 by activated PKCα verified by Phos-tag SDS-PAGE; G-H: Western blot and immunofluorescence detection of key proteins in the NF-κB signaling pathway, Scale bar = 100 μm; I: pNFκB-luc luciferase reporter assay; results are presented as the ratio of firefly luciferase activity to Renilla luciferase activity; J: WB analysis of key NF-κB pathway proteins; K: pNFκB-luc luciferase reporter assay. ∗P < 0.05, ∗∗P < 0.01, ∗∗∗P < 0.001, ∗∗∗∗P < 0.0001 vs. CON group; #P < 0.05, ##P < 0.01, ###P < 0.001, ####P < 0.0001 vs. LPS + ATP or EIF group.

Following further analysis and integration of recent studies on MXRA7, we hypothesized that MXRA7 may interact with PKCα. Detection of PKCα activation levels in the caput and cauda epididymidis of mice in each group (Fig. 7A) revealed that PKCα was significantly activated in the epididymal tissue of EIF mice, and vitamin C partially attenuated PKCα activation. However, no significant difference was observed between the caput and cauda epididymidis. Given that PKCα is a phosphorylating kinase, we speculated that in addition to increasing MXRA7 expression, activated PKCα may also induce MXRA7 phosphorylation, which required further experimental confirmation. We performed Phos-tag SDS-PAGE on MXRA7 from tissue lysates of the caput and cauda epididymidis of each group (Fig. 7B) to separate phosphorylated and non-phosphorylated MXRA7 proteins. The results showed that the phosphorylation level of MXRA7 was significantly increased in the cauda epididymidis of EIF mice, confirming the existence of MXRA7 phosphorylation.

Subsequent in vitro experiments were conducted. PMA is a PKCα agonist that directly activates PKCα with good specificity, while BIM 1 is a specific PKCα inhibitor. In the context of LPS + ATP-induced inflammation and pyroptosis, we added BIM 1 and compared its effects with PMA treatment (Fig. 7C). The results showed that MXRA7 levels were significantly increased by PMA, while BIM 1 markedly inhibited MXRA7 expression, indicating that PKCα upregulates MXRA7 expression levels. Phos-tag SDS-PAGE analysis of MXRA7 from DC-2 cell lysates (Fig. 7D) revealed that MXRA7 phosphorylation was only detected in cells treated with LPS + ATP or PMA, suggesting that MXRA7 phosphorylation is associated with PKCα activation. To further confirm the interaction between PKC and MXRA7, we performed Co-IP assays (Fig. 7E), which showed that p-PKCα had the most prominent interaction with MXRA7. Subsequent in vitro phosphorylation assays of MXRA7 by activated PKCα followed by Phos-tag SDS-PAGE (Fig. 7F) further confirmed the phosphorylation of MXRA7 by activated PKCα. On this basis, we examined the NF-κB signaling pathway (Fig. 7G–I) to verify the effect of PKCα activation on the NF-κB pathway in DC-2 cells. We found that although PKCα activation promoted the activation of IκBα and p65, it exerted a moderate inhibitory effect on p52/p100, which may be related to the action of MXRA7. In contrast, analysis of the NF-κB signaling pathway in PC-1 cells upon PKCα activation (Fig. S6) revealed that the overall activation of NF-κB appeared to be more pronounced, and the effect of PMA seemed greater, which may be attributed to the relative lack of MXRA7 in PC-1 cells.

In addition, since PMA can activate multiple downstream pathways, we combined BIM 1 treatment with MXRA7 knockdown or overexpression to verify the effects of PKCα and MXRA7 on the NF-κB signaling pathway and eliminate interference from unrelated pathways (Fig. 7J–K). The experimental results still confirmed the correlation between PKCα, MXRA7 and NF-κB. Collectively, MXRA7 is mediated and phosphorylated by PKCα, and simultaneously attenuates the activation of the NF-κB signaling pathway by PKCα to a certain extent. However, the specific phosphorylation sites of MXRA7 and the detailed effects of phosphorylation on its protein conformation and function remain to be elucidated, and further follow-up studies are required.

## 4 Discussion

Appropriate exercise promotes human health, enhances physical fitness, prevents and treats various chronic diseases, improves mental state, and elevates quality of life. However, excessive EIF not only hinders physical fitness improvement but also causes adverse reactions in multiple body systems [42]. To date, numerous studies have focused on EIF [[43], [44], [45], [46]], but most have been limited to the musculoskeletal and nervous systems. Obviously, as a systemic multi-system response, EIF may affect various other systems, and its impact on reproduction is multifaceted [2]. In this study, we focused on the relationship between EIF and the reproductive system. By recruiting volunteers with EIF symptoms and comparing their semen and serum samples with those of healthy volunteers, we found serum accumulation of metabolic waste products, decreased antioxidant capacity, significantly elevated oxidative stress and inflammation levels in the reproductive system, reduced seminal epididymal function markers, impaired sperm quality, and decreased sperm motility. Furthermore, EIF-H patients with more severe fatigue not only exhibited a more pronounced decline in sperm motility, but also showed significantly reduced sperm concentration and acrosome integrity, indicating that the degree of sperm damage is positively correlated with the severity of exercise-induced fatigue. This clinical study directly verified that EIF damages the reproductive system through oxidative stress and inflammation [47], and provided preliminary clues for possible epididymal inflammation, laying the foundation for subsequent experiments.

MXRA7 is an autocrine factor. Recent studies have demonstrated its involvement in immunity, inflammation, and cell repair [19,21], and it has been shown to significantly interact with and inhibit inflammatory signaling pathways such as NF-κB [48], suggesting that it may play a role in EIF-induced reproductive system damage and be associated with epididymal inflammation. Transcriptomic analysis using public databases revealed that MXRA7 is highly expressed in epididymitis samples and abundant in epididymal epithelial cells. GSEA analysis further identified its association with multiple inflammatory pathways. Of particular note, there is currently no clinical single-cell transcriptomic database specifically dedicated to EIF-induced epididymal inflammation worldwide, and it is also not feasible to obtain epididymal tissue samples from EIF patients for high-throughput sequencing or mass spectrometry analysis. Therefore, we only used the limited mixed datasets of normal epididymis and general epididymitis available in the GEO database for auxiliary exploratory analysis. In the present study, the expression characteristics of MXRA7 in EIF-induced epididymal inflammation were primarily validated through in vivo and in vitro experiments. Subsequent detection of MXRA7 levels in human semen samples showed a significant increase under EIF conditions. These results confirmed the association between MXRA7 and EIF as well as epididymal inflammation, but the specific mechanism requires further investigation.

Based on the clinical study, we conducted animal experiments for more in-depth research. We first established an EIF mouse model and performed sperm and serum tests similar to those in the clinical study. The results showed that sperm motility was significantly impaired in mice, and sperm concentration and acrosome integrity were also markedly reduced, which was similar to the phenotype observed in EIF-H patients. In addition, we included vitamin C as a supplement to counteract EIF in the animal experiments. Scheduled vitamin C supplementation during exercise is a common strategy for preventing and treating EIF [49] and is widely used in EIF modeling-related studies [24]. The results showed that vitamin C significantly improved EIF symptoms and overall oxidative stress levels, and also enhanced the antioxidant capacity of the reproductive system. However, its effect on alleviating sperm quality impairment was relatively limited, suggesting that it may need to be combined with other drugs that improve sperm quality.

Unlike human volunteers, mouse sperm samples were directly obtained from the epididymis, and epididymal sperm were also significantly damaged. This largely excluded the influence of related organs or tissues such as the prostate and urethra, suggesting that epididymal lesions may be responsible for the sperm damage. Subsequent in-depth studies on the epididymis confirmed that EIF induced oxidative stress and inflammation in the mouse epididymis, and vitamin C had a significant ameliorative effect on these changes. NAG, which can reflect the ability of the epididymis to maintain normal sperm function to a certain extent [36], was also significantly decreased in the epididymis of EIF mice. Further analysis suggested that pyroptosis may also occur in the epididymis of EIF mice. NLRP3, Caspase-1, and GSDMD are the core molecules of pyroptosis, which function in the sequence of “activation-transduction-execution”: NLRP3 acts as an inflammasome sensor that assembles and activates upon stimulation by pathogens or damage signals; activated NLRP3 recruits and activates downstream Caspase-1 [50]; activated Caspase-1 cleaves GSDMD protein, allowing its N-terminal domain to insert into the plasma membrane and form pores, ultimately leading to cell lysis and death, i.e., pyroptosis [[51], [52], [53], [54]]. The results demonstrated that pyroptosis indeed occurred in the epididymis of EIF mice, and pyroptosis may be the dominant mode of cell death, which could be partially reduced by vitamin C intervention. In addition, the protein and mRNA expression levels of MXRA7 in the epididymis were significantly increased under EIF conditions, further confirming its association with EIF and epididymal inflammation. We also found that MXRA7 expression exhibited significant segmental differences in the epididymis, gradually increasing from the caput to the cauda, whereas vitamin C had no statistically significant regulatory effect on MXRA7 expression.

The limited effect of vitamin C on sperm quality despite its effective amelioration of epididymal inflammation and pyroptosis may result from multiple mechanisms. EIF not only triggers epididymal inflammation, but also significantly reduces testosterone levels and impairs testicular spermatogenesis [4,55,56]. EIF-induced sperm damage may occur prior to sperm entry into the epididymis, and vitamin C cannot reverse pre-existing sperm damage. Additionally, the epididymis maintains an independent microenvironment [57] that prevents sperm exposure and subsequent autoimmune reactions. Orally administered vitamin C may struggle to cross the blood-epididymis barrier [58] to directly interact with sperm. In summary, EIF-induced sperm quality decline is a multifactorial process involving systemic metabolic disturbances, reproductive endocrine imbalance, epididymal tissue injury, and direct sperm damage. Vitamin C only targets specific pathological processes and cannot address all damage pathways, but this does not diminish its beneficial effects on other EIF-related pathologies.

Due to differences in cellular composition among different epididymal segments, the epididymis can be roughly divided into three segments: caput, corpus, and cauda [59,60]. We separately examined these three segments of the epididymis in EIF mice and found that oxidative stress and inflammation levels appeared to be higher in the caput than in the corpus and cauda. Further detection revealed that the degree of pyroptosis in the caput was significantly higher than that in the cauda, which was inversely correlated with the expression levels of MXRA7 in different epididymal segments. We therefore hypothesized that the high expression of MXRA7 in the cauda epididymidis may be one of the reasons for its lower inflammation level. Since the caput epididymidis has a more complex physical structure and may suffer greater physical damage such as friction during exercise, we cultured mouse caput epididymal epithelial cells (PC-1) and cauda epididymal epithelial cells (DC-2) in vitro and induced pyroptosis with LPS and ATP [30]. The results still showed that the degrees of inflammation and pyroptosis were lower in DC-2 cells than in PC-1 cells, suggesting that this difference is not solely due to physical structural differences. Meanwhile, MXRA7 expression was also significantly higher in DC-2 cells than in PC-1 cells after LPS and ATP induction, further confirming that MXRA7 may be one of the reasons for the lower inflammation level in the cauda epididymidis.

Subsequently, we performed MXRA7 knockdown and overexpression in the DC-2 cell line. Co-incubation of epididymal sperm from normal mice with conditioned medium from treated DC-2 cells demonstrated the direct effect of MXRA7 on sperm. MXRA7 is also a secretory factor, and its secretion level in the culture medium was positively correlated with its intracellular expression level in epithelial cells, confirming that under inflammatory or other stimulatory conditions, increased MXRA7 expression in epididymal epithelial cells leads to elevated secretion. However, direct intervention with exogenous rMXRA7 on normal spermatozoa or on spermatozoa subjected to inflammatory treatment did not yield statistically significant differences. Meanwhile, the addition of exogenous rMXRA7 did not significantly alter the levels of inflammatory factors in the conditioned medium from which DC-2 cells had been removed, suggesting that MXRA7 does not directly interact with inflammatory factors. This may indicate that the protective effect of MXRA7 on spermatozoa is primarily achieved by alleviating epididymal epithelial cell inflammation. Whether MXRA7 secreted by cauda epididymis epithelial cells can directly sustain sperm motility still requires further in-depth investigation.

After literature search and analysis, we hypothesized that MXRA7 may be associated with the NF-κB signaling pathway. Examination of the NF-κB signaling pathway in the caput and cauda epididymidis of mice revealed that the NF-κB pathway was significantly activated in the epididymis of EIF mice, and the degree of activation in the cauda was significantly lower than that in the caput. Subsequent in vitro experiments showed that MXRA7 knockdown in DC-2 cells significantly increased both inflammation levels and NF-κB activation, while MXRA7 overexpression inhibited the LPS and ATP-induced increases in inflammation and NF-κB activation. These results confirmed that MXRA7 reduces epididymal inflammation and pyroptosis of cauda epididymal epithelial cells by inhibiting the NF-κB signaling pathway (including both canonical and non-canonical pathways).

It was found through MXRA7-related experiments that MXRA7 expression was significantly upregulated under EIF conditions. In vivo experiments showed that vitamin C intervention could effectively ameliorate EIF-induced inflammation and tissue damage, while it did not promote MXRA7 expression and even exhibited a certain degree of inhibition. In vitro experiments revealed that vitamin C intervention did not affect MXRA7 levels in normally cultured DC-2 cells, but could reduce MXRA7 levels in LPS- and ATP-induced DC-2 cells as the degree of inflammation decreased. However, subsequent experimental results from gene intervention and sperm incubation all demonstrated the anti-inflammatory effect of MXRA7. This may suggest that MXRA7 is an injury-induced endogenous protective molecule, and its upregulated expression is not a driver of injury, but an active protective response initiated by the body in the face of EIF-induced oxidative stress, inflammation and pyroptotic damage. Since vitamin C is an exogenous intervention agent that directly eliminates upstream damage, it can remove the upstream induction signals that promote MXRA7 expression by scavenging oxidative stress and alleviating inflammation, thereby reducing MXRA7 from compensatory overexpression back to the physiological baseline level. Therefore, vitamin C does not have a direct inhibitory effect on MXRA7. For example, the well-recognized endogenous antioxidant SOD [61] is compensatorily elevated during oxidative stress/inflammatory damage; when effective antioxidant/anti-inflammatory intervention is given to eliminate the damage, the expression of SOD will decrease with the alleviation of injury. MXRA7 is also such an endogenous protective molecule.

PKC is a family of calcium- and phospholipid-dependent protein kinases that are widely involved in the regulation of cell proliferation, differentiation, apoptosis, inflammatory responses, and signal transduction [62]. The PKC family includes multiple isoforms, and PKCα, a classical PKC isoform, can regulate NLRP3 inflammasome activation, pyroptosis progression, and epithelial cell function [[63], [64], [65]]. It is directly associated with the NF-κB signaling pathway and can activate the canonical NF-κB pathway [66]. Combined with recent studies [21], we hypothesized that MXRA7 may be closely related to PKC and conform to the characteristics of a PKC downstream molecule. In vivo and in vitro experiments demonstrated that MXRA7 was not only directly affected by PMA (a PKCα agonist [64]) and BIM 1 (a PKCα inhibitor [67]), but also was present in a phosphorylated form in response to inflammatory stimulation or EIF. In vitro kinase assays and co-immunoprecipitation (Co-IP) of MXRA7 and PKC confirmed that activated PKCα phosphorylates MXRA7. However, it must be acknowledged that since PKC can also affect MXRA7 expression levels, the specific mechanism of phosphorylated MXRA7 requires further investigation. However, it must be acknowledged that since PKC can also affect MXRA7 expression levels, the specific mechanism of action and functional changes of phosphorylated MXRA7 require further investigation. Moreover, the failure to identify the key phosphorylation sites of MXRA7 represents one of the major limitations of this study. In addition, it was also found that PKCα exerts a dual regulatory effect on the NF-κB pathway—not only directly activating the classical pathway but also indirectly inhibiting both the classical and non-canonical pathways through MXRA7. However, the balance of this regulatory network requires in-depth analysis, which will be a key focus of future research.

In summary, MXRA7 can inhibit the NF-κB pathway. Under external oxidative stress and inflammatory stimulation, activated PKCα activates the canonical NF-κB pathway while simultaneously inhibiting both the canonical and non-canonical NF-κB pathways through MXRA7, which may represent a negative feedback mechanism. The presence of MXRA7 reduces the transcription of NLRP3 by activated NF-κB, thereby decreasing inflammation and pyroptosis.

## 5 Conclusion

This study demonstrated that EIF induces oxidative stress and inflammation in the epididymis, thereby triggering pyroptosis of epididymal epithelial cells and eventually impairing sperm quality. Notably, the severity of inflammation and pyroptosis declined progressively from the caput to the corpus and cauda epididymidis. The present study identified MXRA7 as one of the endogenous protective molecules in the epididymis. Its high expression in cauda epididymal epithelial cells attenuates inflammatory responses and cellular pyroptosis by inhibiting the NF-κB signaling pathway. Meanwhile, both the expression and phosphorylation levels of MXRA7 are mediated by PKCα, and MXRA7 in turn attenuates PKCα-mediated activation of the NF-κB signaling pathway.

Collectively, this study reveals a novel molecular mechanism underlying EIF-induced male reproductive damage, provides MXRA7 as a potential target for the prevention and treatment of exercise-induced reproductive injury, and also partially explains the molecular basis for the differential inflammatory susceptibility of different epididymal segments. Future studies can further identify the key functional phosphorylation sites of MXRA7, perform in-depth analysis of the dual regulatory role of PKCα on the NF-κB pathway with the involvement of MXRA7, develop or screen specific intervention agents targeting MXRA7, and validate their translational application value in clinical populations with exercise-induced reproductive damage.

## Ethics approval

Regarding studies involving human subjects: This study was approved by the Institutional Ethics Committee (Approval No.: HBZY2023-C10-02). All experiments were performed in accordance with the principles of the Declaration of Helsinki of the World Medical Association, and informed consent as well as voluntary cooperation were obtained from each participant; Regarding animal experiments: All experimental procedures were strictly performed in accordance with the Guidelines for the Care and Use of Laboratory Animals and were approved by the Institutional Animal Care and Use Committee of Hubei University of Chinese Medicine (Approval No.: HUCMS55714520).

## Clinical trial number

Not applicable.

## Data availability statements

The data underlying this article will be shared on reasonable request to the corresponding author.

## Funding

This work was supported by grants from Innovation and Development Joint Fund Project of 10.13039/501100003819Hubei Natural Science Foundation (2026AFC0838); Innovation and Development Joint Fund Project of 10.13039/501100003819Hubei Natural Science Foundation (2024AFD259); Innovation and Development Joint Fund Project of 10.13039/501100003819Hubei Natural Science Foundation (2025AFD577); Science and Technology Special Project of the 10.13039/501100005891State Administration of Traditional Chinese Medicine (GZY-KJS-2025-011).

## CRediT authorship contribution statement

**Kunyang Tang:** Conceptualization, Writing – original draft, Writing – review & editing. **Xiaocui Jiang:** Conceptualization, Writing – review & editing. **Yanyan Zhou:** Formal analysis, Writing – review & editing. **Xin Hu:** Formal analysis, Methodology. **Jiasen Liu:** Funding acquisition, Methodology. **Donghui Huang:** Investigation, Resources. **Xiaoming Yu:** Supervision. **Min Zhao:** Validation. **Ying Liu:** Visualization. **Jigang Cao:** Data curation, Resources, Software. **Zhipeng Fang:** Funding acquisition, Project administration, Resources. **Min Xiao:** Conceptualization, Resources, Writing – review & editing.

## Declaration of competing interest

On behalf of all authors, the corresponding author states that there is no conflict of interest. Certain graphical elements used in some figures of this study were sourced from Freepik (https://www.freepik.com), utilized under a free license with mandatory attribution.
