# Supplementary material for: MXRA7 attenuates sperm injury from exercise-induced fatigue via suppression of epididymal inflammation
Source: Redox Biol. 2026 Jul 14;96:104301. doi: 10.1016/j.redox.2026.104301 (PMC13393802; doi:10.1016/j.redox.2026.104301)
Supplement: Multimedia component 1 [file mmc1.docx]

**Supplementary Material**

**1 Supplementary Fig.S1**


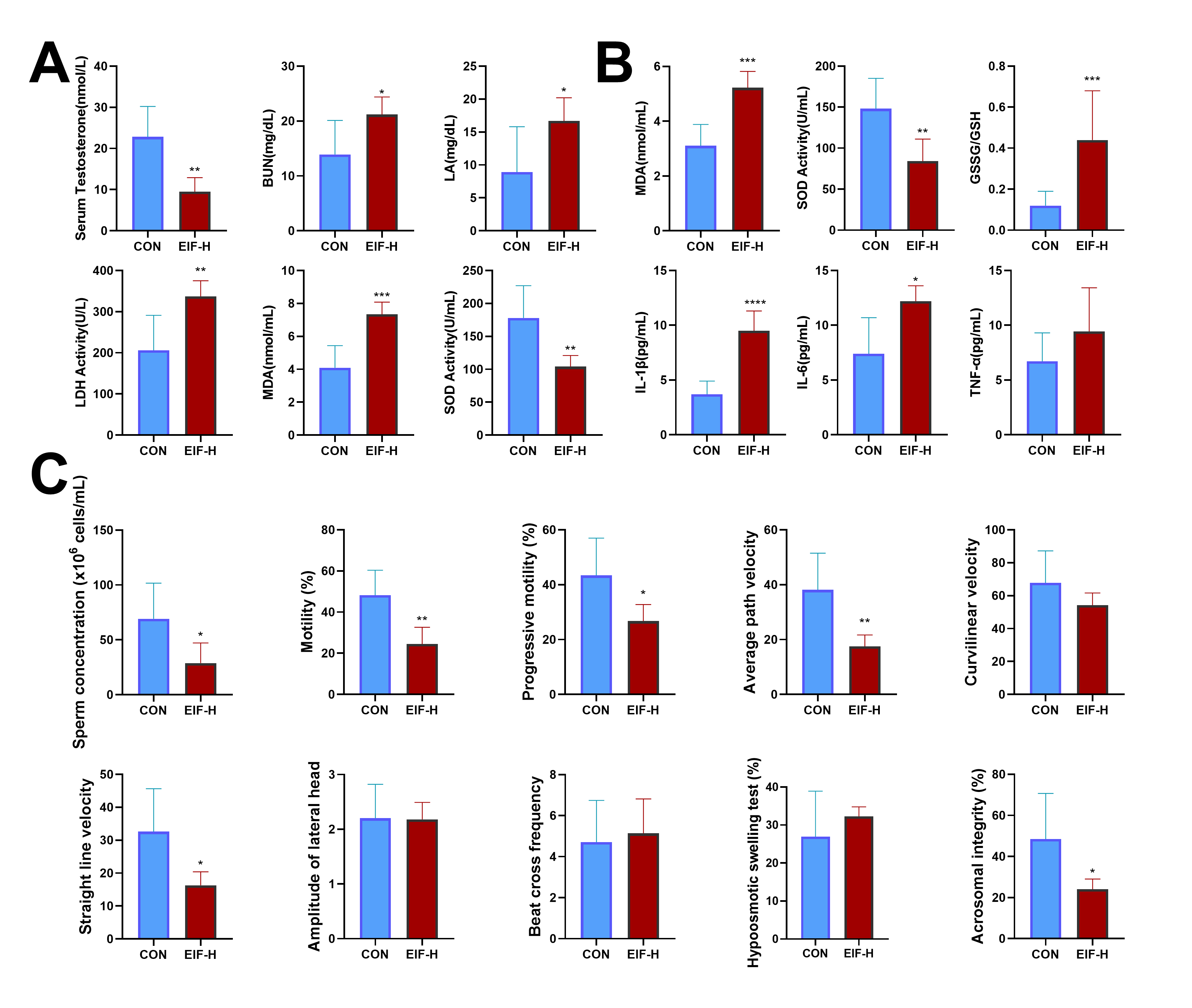


Fig.S1 Parameters of serum, semen, and sperm in EIF-H patients

Note: A: Serum levels in EIF-H patients; B: Levels of oxidative stress markers and inflammatory cytokines in seminal plasma; C: Sperm quality parameters, acrosome integrity, and hypoosmotic swelling test results. *P < 0.05, **P < 0.01, ***P < 0.001, ****P < 0.0001 vs. CON group.

**2 Supplementary Fig.S2**


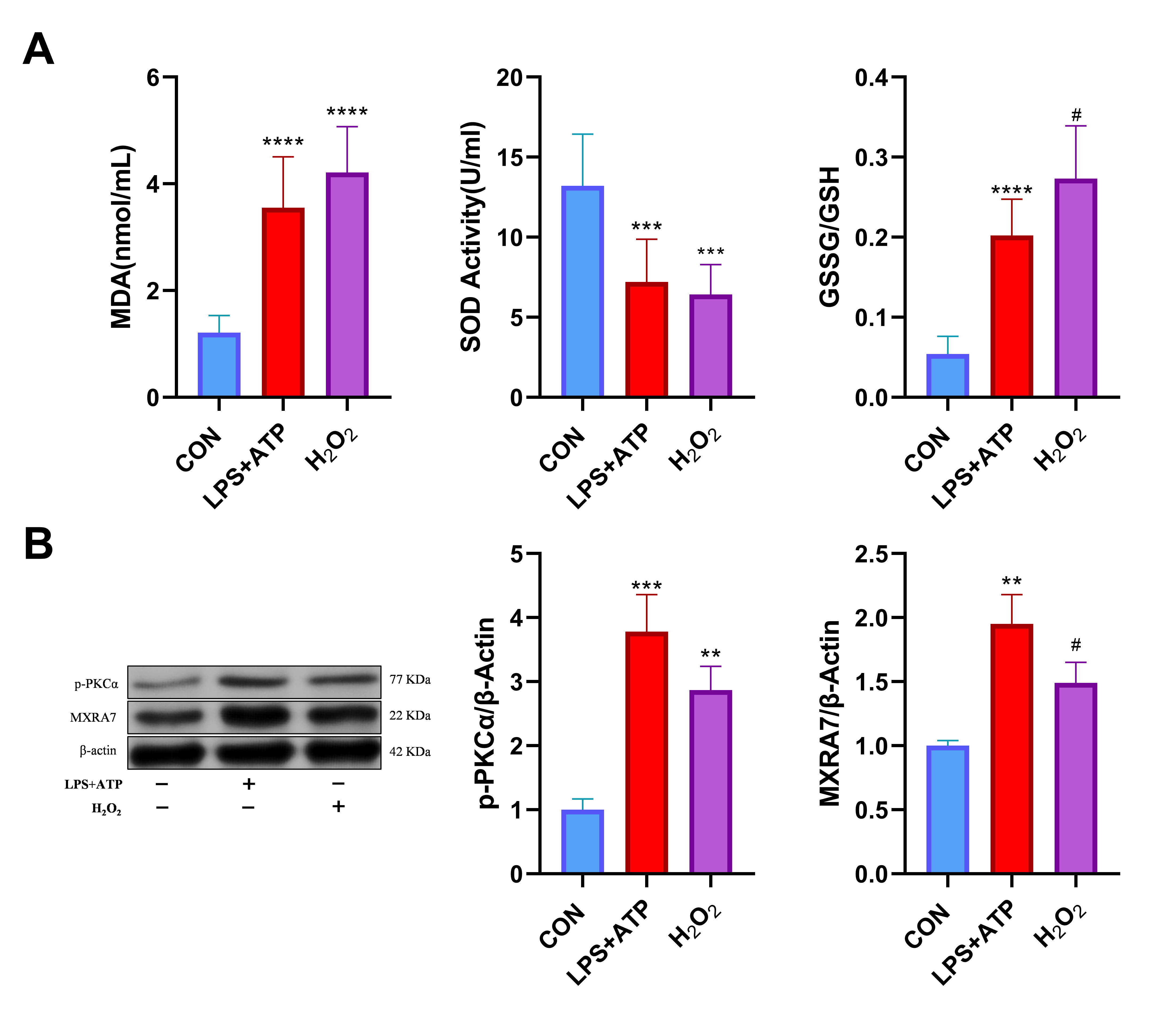


Fig.S2 Differences between LPS+ATP and H₂O₂ cell models in this study

Note: A: Oxidative stress markers in cell supernatants; B: Western blot analysis of MXRA7 and p-PKCα expression levels. *P < 0.05, **P < 0.01, ***P < 0.001, ****P < 0.0001 vs. CON group; #P < 0.05, ##P < 0.01, ###P < 0.001, ####P < 0.0001 vs. LPS+ATP group.

**3 Supplementary Fig.S3**


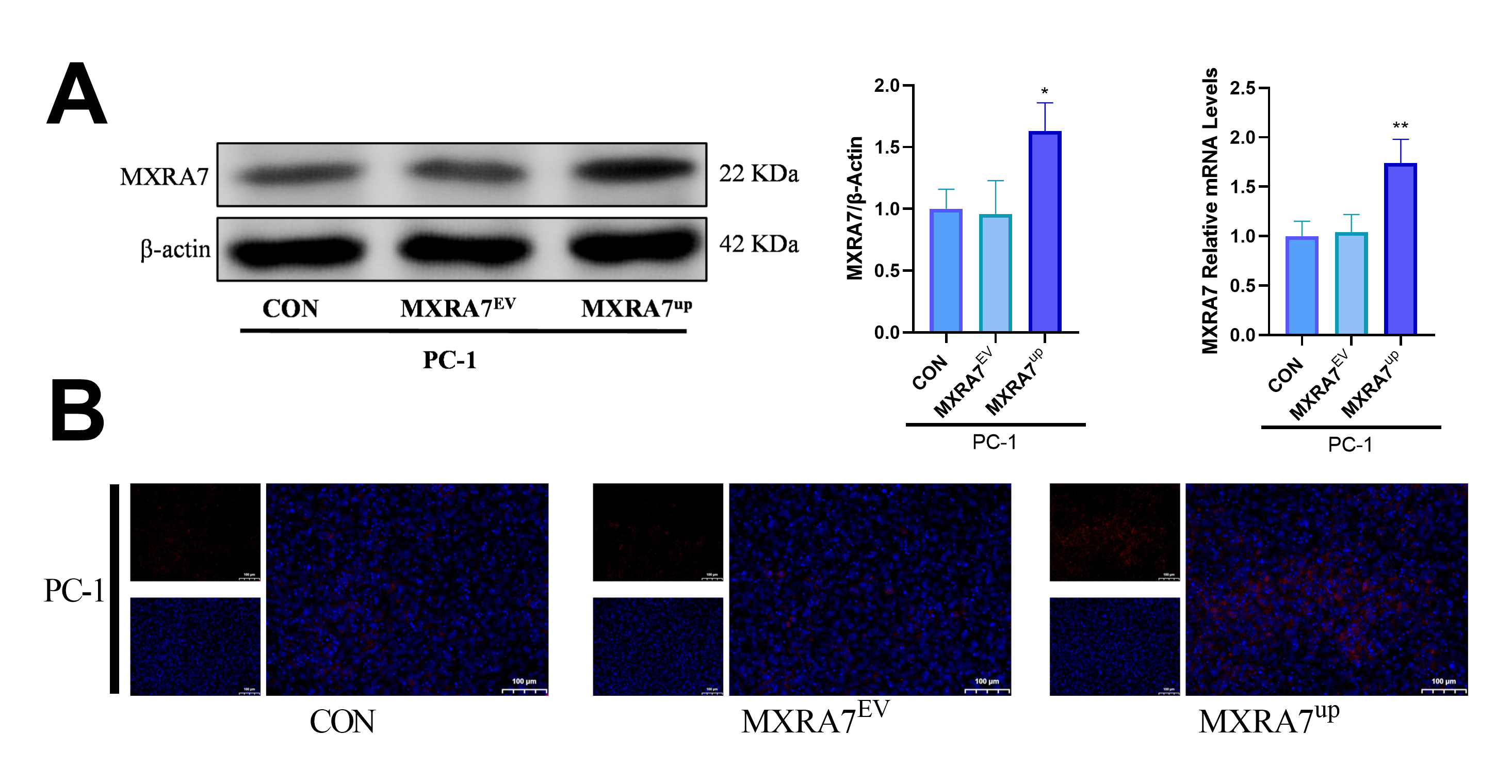


Fig.S3 MXRA7 overexpression in PC-1 cell line

Note: A: Protein expression and mRNA levels of MXRA7; B: MXRA7 immunofluorescence in PC-1 cell line, Scale bar = 100 μm. *P < 0.05, **P < 0.01 vs. MXRA7^EV^ group.

**4 Supplementary Fig.S4**


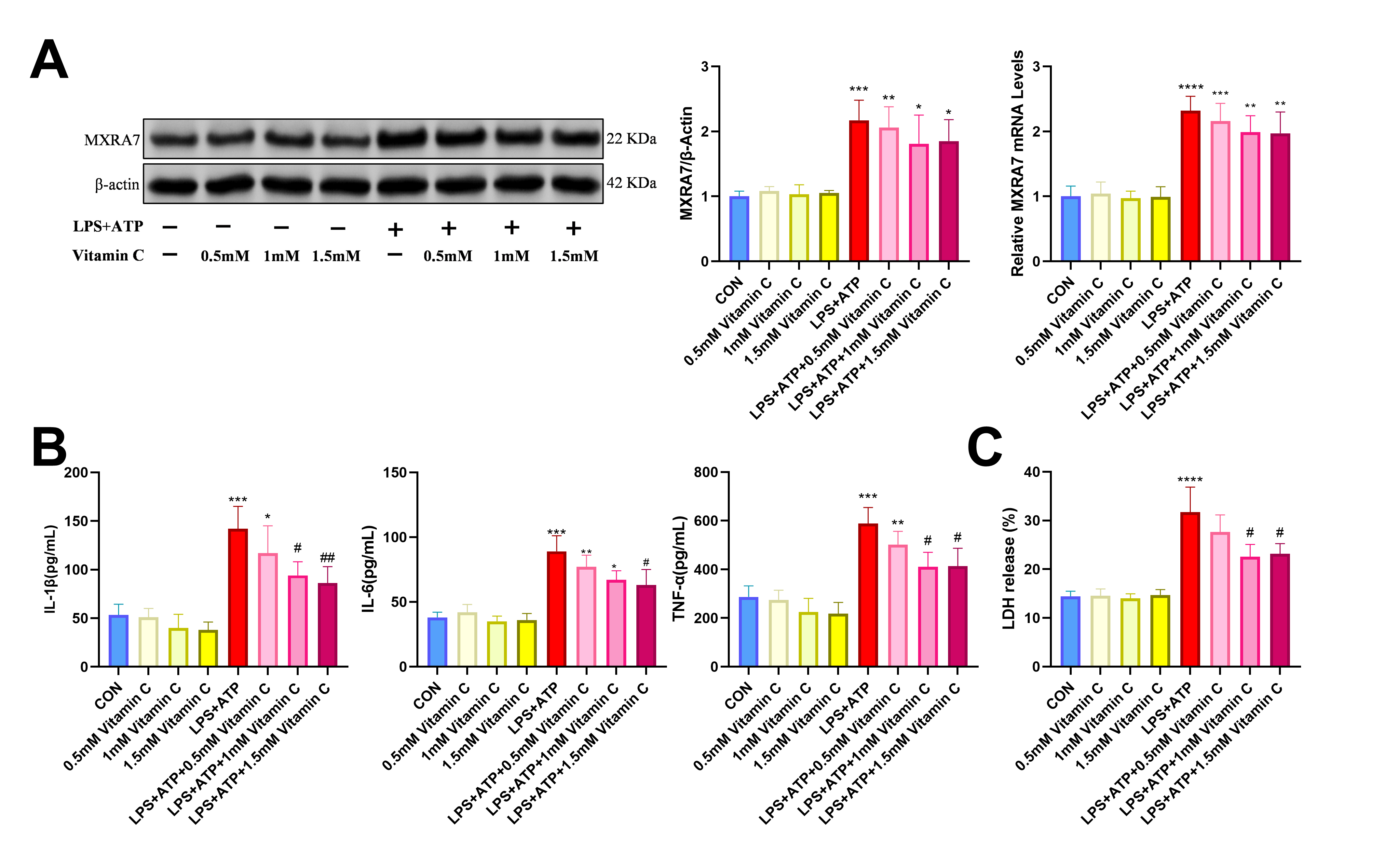


Fig.S4 DC-2 cells treated with gradient doses of vitamin C

Note: A: Protein expression and mRNA levels of MXRA7; B: Levels of inflammatory cytokines in the cell supernatant; C: Cellular pyroptosis level detected by LDH release assay. *P < 0.05, **P < 0.01, ***P < 0.001, ****P < 0.0001 vs. CON group; #P < 0.05, ##P < 0.01, ###P < 0.001, ####P < 0.0001 vs. LPS+ATP group.

**5 Supplementary Fig.S5**


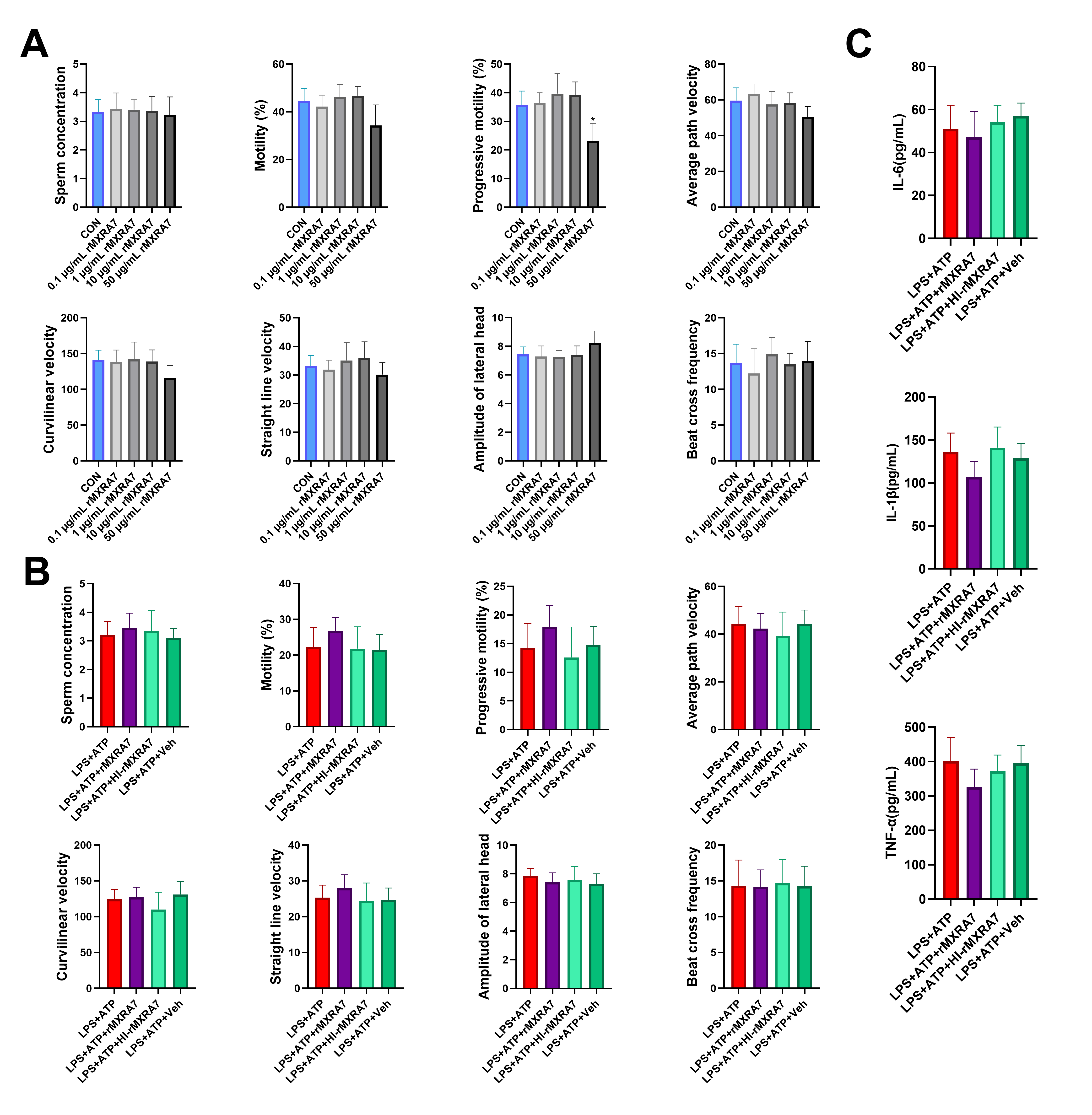


Fig.S5 Exogenous recombinant MXRA7 intervention on spermatozoa in vitro

Note: A: Changes in sperm parameters under different doses of rMXRA7; B: Changes in sperm parameters under inflammatory conditions, with rMXRA7 at a dose of 10 μg/mL, HI-rMXRA7 being heat-inactivated rMXRA7, and Veh being the vehicle of rMXRA7; C: Levels of inflammatory factors in the supernatant. *P < 0.05 vs. CON group.

**6 Supplementary Fig.S6**


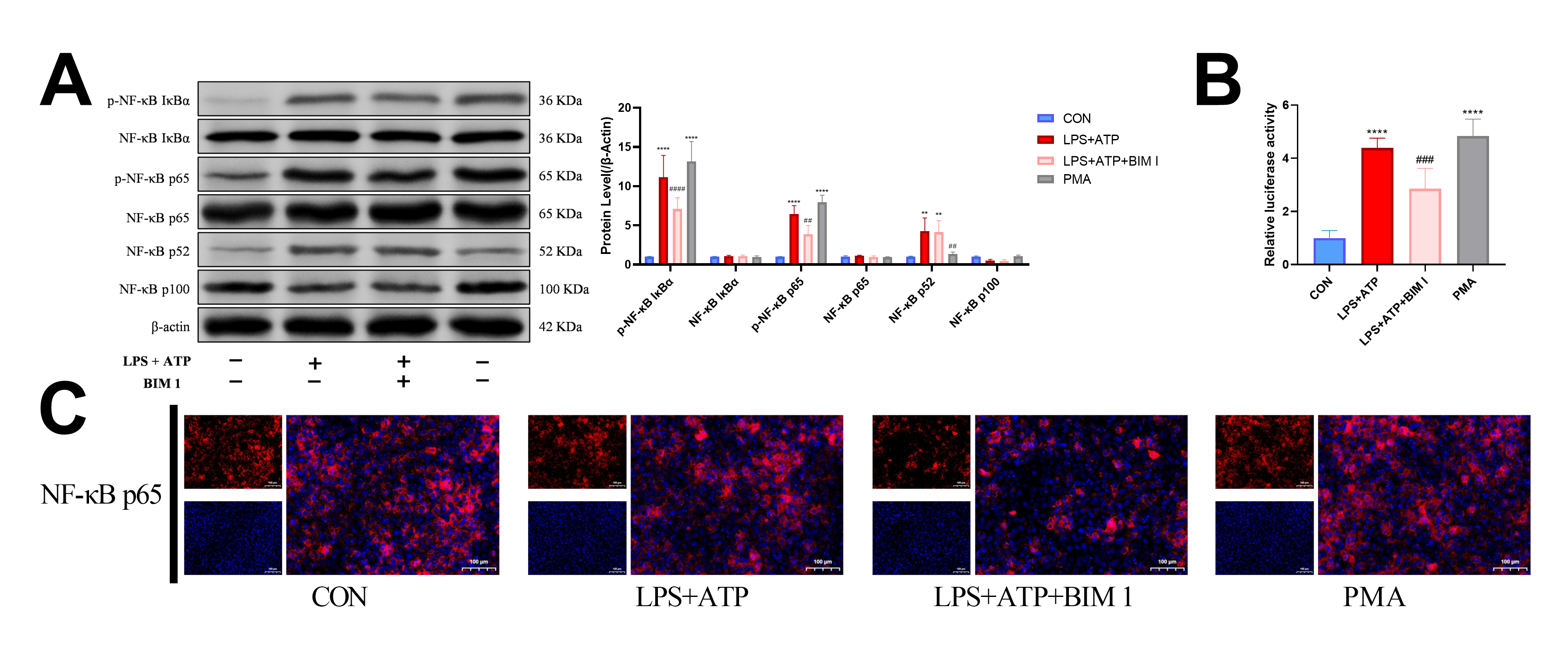


Fig.S6 Effect of PKCα activation on NF-κB in PC-1 cells

Note: A: Western blot detection of key NF-κB pathway proteins; B: pNFκB-luc luciferase reporter assay, results expressed as the ratio of firefly luciferase activity to Renilla luciferase activity; C: Immunofluorescence staining of P65. *P < 0.05, **P < 0.01, ***P < 0.001, ****P < 0.0001 vs. CON group; #P < 0.05, ##P < 0.01, ###P < 0.001, ####P < 0.0001 vs. LPS+ATP group.
